# Microstructural Modeling and Simulation of a Carbon Black-Based Conductive Polymer—A Template for the Virtual Design of a Composite Material

**DOI:** 10.1021/acsomega.2c01755

**Published:** 2022-08-11

**Authors:** Yuanzhen Wang, Chensheng Xu, Timotheus Jahnke, Wolfgang Verestek, Siegfried Schmauder, Joachim P. Spatz

**Affiliations:** † Department of Cellular Biophysics, Max Planck Institute For Medical Research, Jahnstraße 29, 69120 Heidelberg, Germany; ‡ Institute For Molecular Systems Engineering (IMSE), Heidelberg University, Im Neuenheimer Feld, 69120 Heidelberg, Germany; § Institute For Materials Testing, Materials Science And Strength Of Materials (IMWF), University Of Stuttgart, Pfaffenwaldring 32, 70569 Stuttgart, Germany

## Abstract

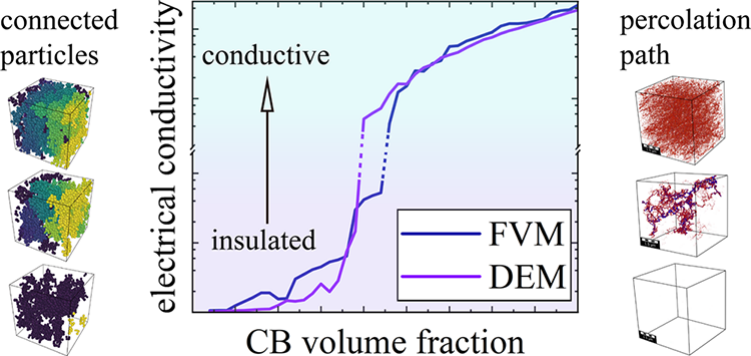

Carbon black is the most frequently applied conductive additive in rubber and polymer composites. In this work, we show how a carbon black microstructure in a polymer matrix can be conclusively modeled based on carbon black aggregation as well as an agglomeration mechanism using a state-of-the-art mathematical model. This novel and flexible microstructural modeling method enables us to virtually investigate the morphology of conductive additives within a polymer matrix and can be adapted to many conductive polymer combinations used for different applications. Furthermore, we calculate the electrical conductivity of the composite using a finite volume-based as well as a discrete element-based simulation technique and validate the results with experimental data. Utilizing a novel discrete element method (DEM) modeling technique, we were able to improve calculation times by a factor of 12.2 compared to finite volume method (FVM) simulations while maintaining high accuracy. Using this approach, we are able to predict the required carbon black content and minimize the amount of additive to create a polymer composite with a designated target conductivity.

## Introduction

Conductive additives are used in a great number of applications—for conductive coatings,^1,2^ three-dimensional (3D) printing,^2^ or as a mechanical and electrical connector in energy storage devices, especially lithium-ion batteries.^3,4^ Lithium ion batteries comprise two electrodes, each one consisting of a current collector that provides electronic conductivity and an active material layer that partakes in the electrochemical reaction.^5−7^ The most common active materials, especially cathode-active materials like LiNiMnCoO_2_ (NMC), are available in the form of a powder and as such, are neither mechanically nor electrically cohesive.^8,9^ To render the active material layer stable and conductive, a combination of binder, e.g., Sodiumcarboxymethylcellulose (NA-CMC) or poly(vinylidene fluoride-*co*-hexafluoropropylene) (PVDF-HFP) and conductor-like carbon black (CB) are used.^10^

A carbon black particle has an isotropic electrical conductivity of approximately 4000 S m^–1^, and its primary spherical particle diameter ranges from 10 to 100 nm.^11,12^ Partially graphitic carbon black primary particles tend to form aggregates and agglomerates due to their large specific surface area. Pure binders (e.g., PVDF-HFP/CMC, which are used to improve the mechanical connection strength) are highly insulating materials (σPVDF < 10^–8^ S m^–1^). To achieve conductivity, a conductive pathway needs to be established within the polymer by carbon black particles. Therefore, the ratio between carbon black and the binder plays an essential role in the conductivity of the conductive binder phase (CBP), the mixed phase of conductive additive, and the binder used for coating electrodes.

**Figure 1:**
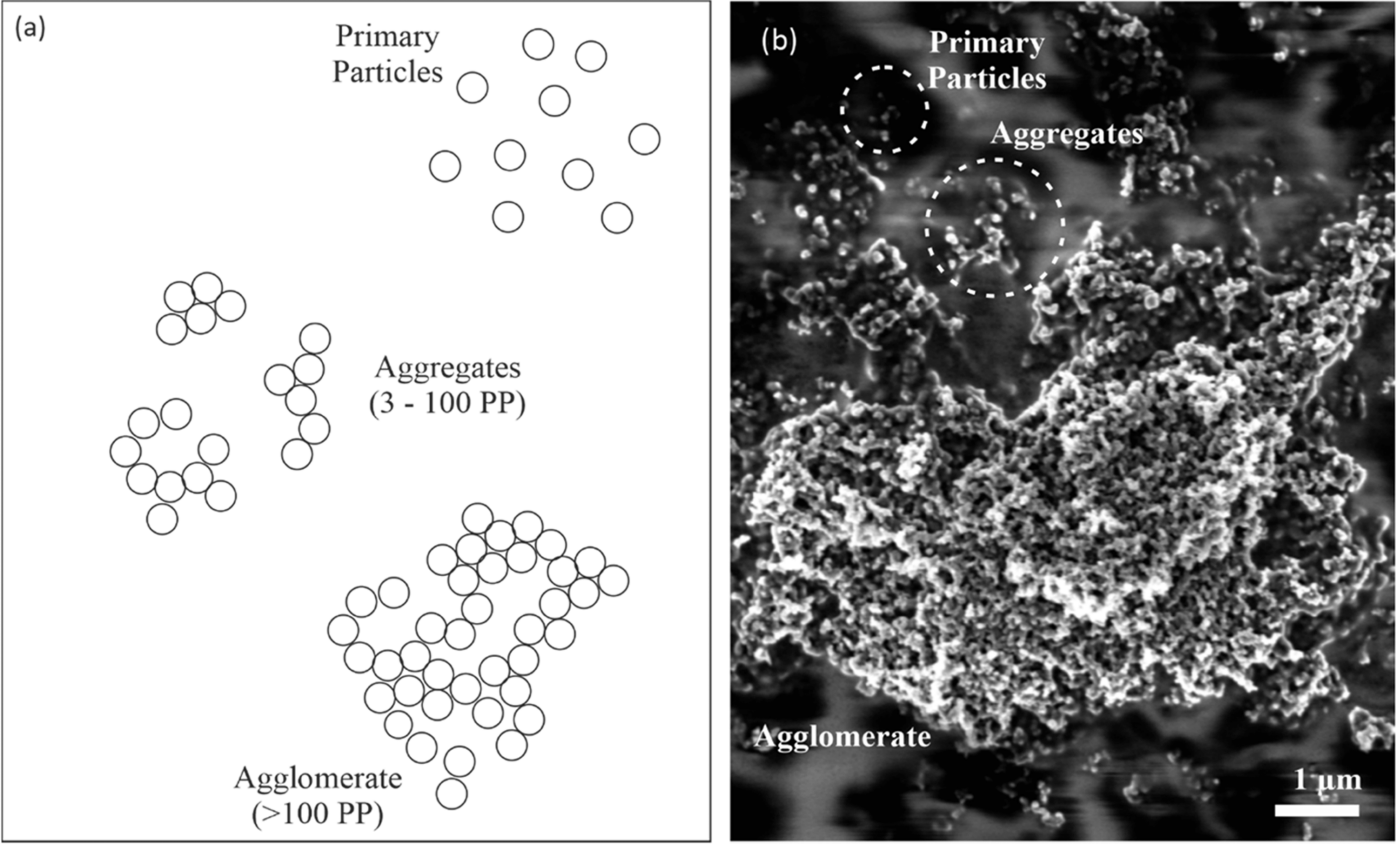
CB agglomerates. (a) Schematic illustration, (b) scanning electron microscopy (SEM) micrograph of carbon black particles embedded in PVDF-HFP.

As a result, the carbon particle size, the volume fraction of CB, as well as its aggregation and agglomeration mechanisms^13^ (see Figure 1) have a significant impact on the electrical conductivity of the conductive binder phase. The agglomeration mechanism is mostly governed by the interaction between the CB particles themselves (their tendency to agglomerate) and their interaction with the surrounding polymer matrix. A description of the process of aggregate formation and the electron conductivity between closely connected aggregates was first conclusively shown by Balberg.^14^ He was able to show that inter-particle tunneling effects lead to a large increase in conductivity between the carbon black aggregates. This finding was able to explain differences in the results obtained using the classical percolation theory model and experimental results for percolation thresholds. The microstructure formed by the assembled agglomerates (i.e., their distribution) relates strongly to the conduction pathway and the percolation threshold.

The effect of how aggregates assemble on the morphology of polymer blends was investigated experimentally by Sumita et al.^15^ They were able to show that the surface tension of the polymer plays an important role in the formation of the respective microstructure. Consequently, the formation of the percolation path in such polymer composites is highly dependent on its components. For instance, Ou et al.^16^ compared a mechanical and a solution-based mixing method, resulting in ordered and randomly distributed carbon black particles within the microstructure. They found that the percolation path in the ordered microstructure is formed at 0.26 vol % CB content, whereas, in comparison, the percolation path in the case of the unordered microstructures forms at 2.7 vol % CB.^16^

Nevertheless, carbon black/polymer composites have been intensely studied on both the microscale and the macroscale but usually exclusively on an empirical basis, which requires a huge number of experiments. This has, up to now, hindered researchers from further optimizing the composite and adapting their studies to other conductive polymer systems. To unify the nanoscopic approach of the aggregate’s internal conductivity with the macroscale distribution of the aggregates within the polymer (usually obtained by time-consuming experiments), this study focuses on the connection between CB single particles and aggregate formation as well as investigating their distribution in the polymer matrix. To model the microstructure of the aggregates as well as their distribution, a two-step modeling approach is applied.

(i) Different CB aggregates are statistically modeled and compared with experimentally obtained CB aggregate structures (based on a model by Balberg).^14^
(ii) The aggregates (or their simplified counterparts) are randomly distributed in the polymer.

With this approach, the virtual investigation, design, and optimization of the CB-polymer become possible. Worthwhile mentioning is that the CB aggregates and polymer matrix interaction can influence the uniformity of the CB aggregates distribution.^15^ However, with regard to the practical application in battery research, the dispersion rotating speed is ultrahigh (10,000 rpm), and the dispersing time is up to 2 h to ensure a random and homogeneous dispersion and less agglomeration. Thus, in (ii), the aggregates are set to be randomly distributed, regardless of the polymer’s impact. Figure S1 shows that CB particles distribute quite homogeneously in both PVDF and the CMC matrix.

A main goal of this work was the simulation of the conductivity using a mesh-free discrete element method (DEM) on the basis of particle principles and comparing it to a simulation on the basis of a finite volume method (FVM), while both calculations are validated with experimental measurements.

## Experimental Section

### Sample Preparation

Super P conductive carbon black (Alfa Aesar, 99+% trace metals basis, CAS: 1333-86-4) and PVDF-HFP (Sigma-Aldrich, CAS: 9011-17-0) were used as conductive agent and binder, respectively. PVDF-HFP binder was mixed with acetone (Alfa Aesar, 1 wt % EMK) at different mass ratios in a glass beaker. The mixture was stirred at room temperature with a magnetic stirrer (IKA Model RET basic) until the PDVF-HFP was fully dissolved. Meanwhile, carbon black was weighed and added to the PVDF-HFP/acetone mix and dispersed with a disperser (IKA T25 Ultra Clean) at 6000 rpm for 10 min. The mixture composition is shown in Table 1. To remove the bubbles from the sample, the mixture was immersed in an ultrasonic cleaning bath (VWR ultrasonic cleaner) for 15 min. Afterward, the mixture was coated on a copper foil, and the doctor-blading technique was applied to control the thickness of the coating.^17^

**Table 1:** Mixture Composition During Sample Preparation

composition	density	amount	wt %	vol %
CB	1.89 g cm^–3^	0.1–0.5 g	5–50%	4.7–48.5%
PVDF-HFP	1.78 g cm^–3^	0.5–1.8 g	50–95%	51.5–95.3%
acetone	0.784 g cm^–3^	15 mL	*X*	*X*

### Electrical Conductivity Measurement

Because of its high reproducibility and the possibility to measure the conductivity of arbitrarily shaped samples, the van der Pauw method was used as the method for measuring electrical conductivity. To validate the method, the thickness of the sample must be much smaller than the width and length of the sample. Thus, the doctor-blading thickness was set to 300 μm during coating. The final thickness of the coating after the drying process was approximately 35 μm. The CB and the polymer samples were removed from the copper foil and cut into the shape shown in Figure S2. This specific sample shape was chosen because a symmetrical shape is preferable, and the grooves help to position the electrodes of the 4-point source meter (Keithley 2615 A Source meter) when switching the current source and voltage meter. The four electrode positions are marked with the numbers 1–4 in Figure S2. Resistance values were obtained by repeating resistance measurements after switching the polarities of both the current source and the voltage meter to achieve a more reliable and homogenous conductivity value. That way, any offset voltage, such as thermoelectric and contact resistance-induced voltage, is canceled out. The sheet resistance, and thus the electrical conductivity, was determined using the van der Pauw formula.^39^

### Computational Methods

For modeling the morphology of the carbon black aggregates, a two-step approach was applied. First, MATLAB 2019A was used to determine the position and size of the CB primary particles, which then fuse together and form CB aggregates. Large amounts of CB aggregates in different sizes were modeled during this step. Second, the random distribution of the carbon black aggregates was realized using Geodict, a simulation software developed by Math2Market. Each CB aggregate was randomly allocated a position and a rotation angle inside the polymer matrix. Meanwhile, a collision removal algorithm was applied to account for CB aggregates overlapping. Detailed information is given in the Supporting Information (S1).

Separately, based on Ohm’s law and Kirchhoff’s law, two simulation methods were carried out to calculate the electrical conductivity and to visualize the percolation path. Ohm’s law was implemented with a finite volume method (FVM) in Geodict; The discrete element method (DEM) was implemented with the open-source software Python. Detailed information is given in the Supporting Information S1.

**Figure 2:**
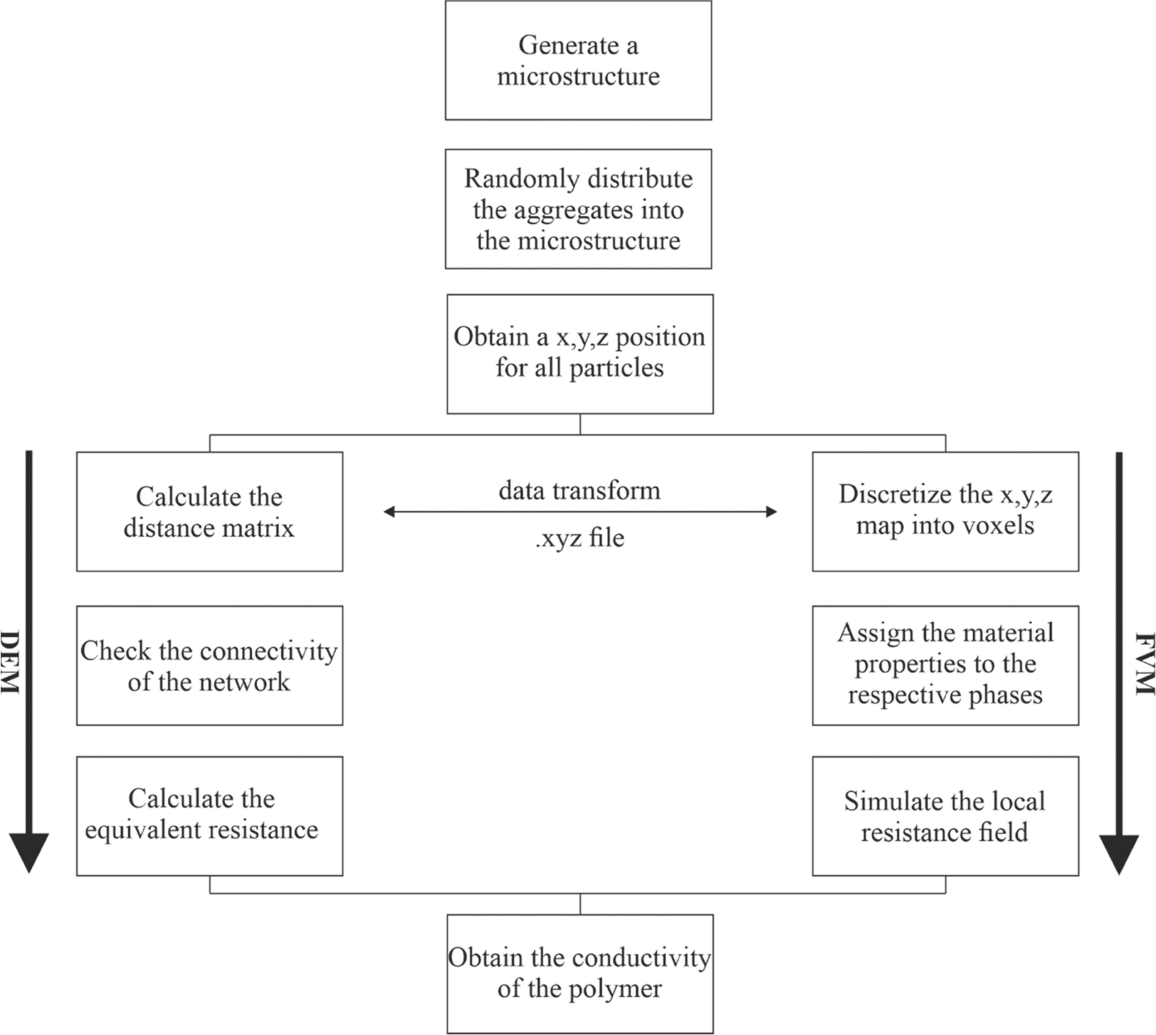
Schematical flowchart of the simulation procedure used to simulate the conductivity of a polymer. Based on the same microstructure, a different approach to simulate the electrical conductivity is selected, and its results are compared to each other and the experiment.

Figure 2 schematically shows the flowchart of the modeling and simulation of the microstructures. The modeled microstructure was then used to calculate and simulate the electrical conductivity. This was done (i) using a recursive function solver directly on the voxel-based microstructure and (ii) comparing it to the results of the DEM technique by making use of the particle-based topological structure of the aggregate distribution. Finally, the electrical conductivity was calculated using Kirchhoff’s law.

## Results and Discussion

### CBP Morphological Modeling

**Figure 3:**
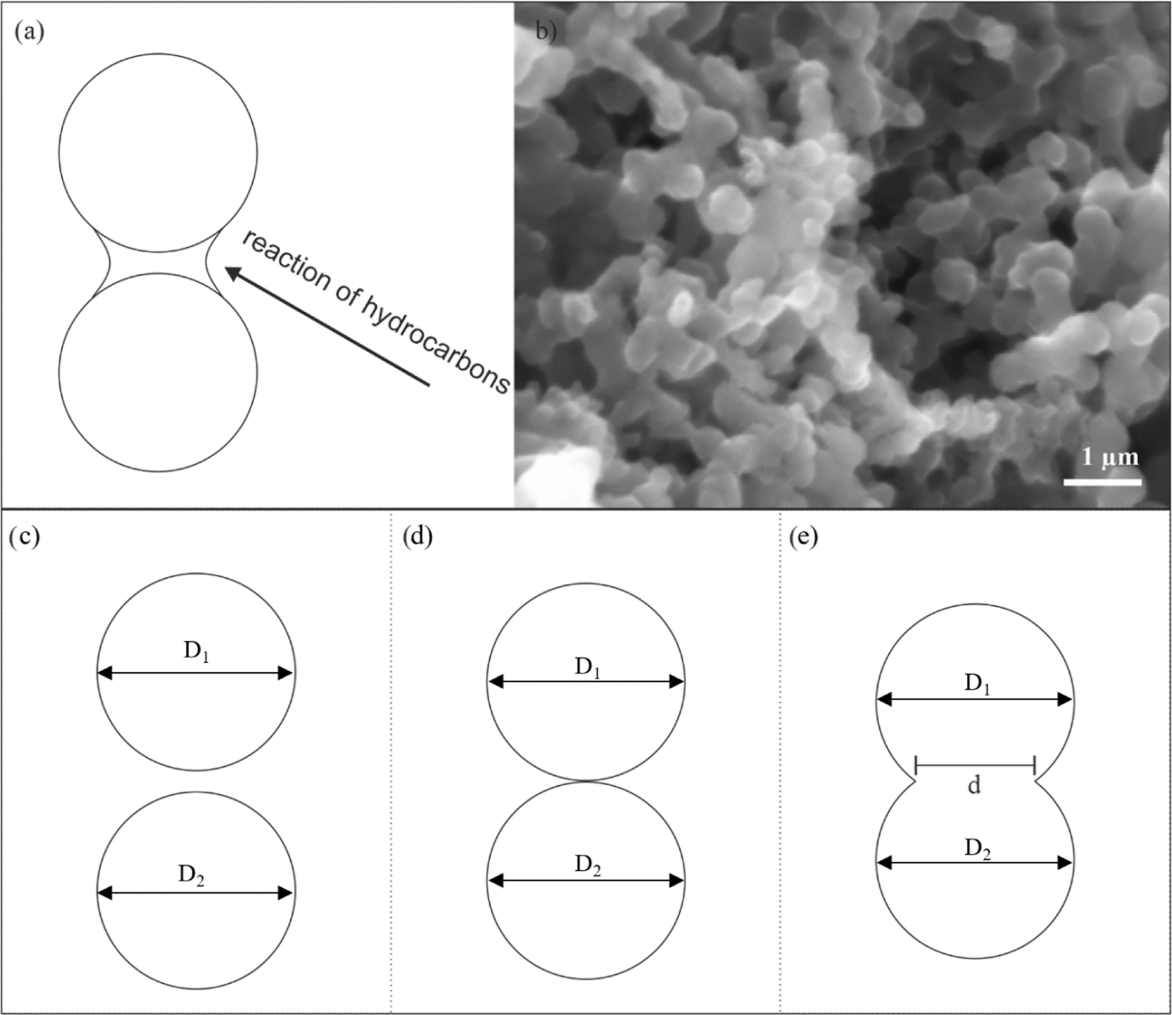
Carbon black aggregation and agglomeration. (a) Surface growth at the inter-particle bonding neck of two connected CB primary particles during production; (b) CB aggregation results in a combination of elongated chains of CB particles and clusters, creating an overall porous microstructure; (c) two-particle model illustration, two CB particles approach each other and (d) establish contact; and (e) equilibrium contact between two electrically conductive, smooth elastic spheres with a given surface energy γ and Young’s modulus *E*.

Carbon black aggregates are composed of spheroidal “primary particles” strongly fused together to form discrete entities.^18^ During the formation, several primary CB particles fuse to form aggregates that are usually arranged in a chain-like manner or as clustered carbon black agglomerates (see Figure 3b).

According to experimental studies, primary CB particles in a CB powder or in a polymer composite do not exist as isolated particles; instead, they form aggregates with a size between 50 and 3000 nm (depending on the number of primary particles and their assembly). Both aggregate size and structure depend strongly on the surface and interface tension of the CB primary particles and the production process of the carbon black particles.^19^ Studies show that aggregates undergo fusion during production, a process described by Lahaye et al.^20^ During the typical production process of carbon black particles, hydrocarbons undergo thermal decomposition at a defined temperature under the exclusion of oxygen.^21,22^ Upon reaching the decomposition temperature, carbon black clusters form in the gas phase and then grow into spherical primary particles. These primary particles then form aggregates in the gaseous environment in which they are able to move randomly. Since residual hydrocarbon precursor is still present at this phase of the reaction process, these particles are fused together. This is caused by acetylene reacting with carbon on the neck-like connection between the aggregates, resulting in surface growth (see Figure 3a).^20^ To capture this in our simulations, carbon black aggregates are modeled as overlapping spheres. CB aggregates form an electrical network composed of junctions and branches within the microstructure. Next, the resulting data on aggregate formation and distribution was correlated with the respective physical properties like surface tension and surface area of the carbon black aggregates. Aggregate modeling was performed based on data from the statistical analysis looking at the aggregate size and the number of primary particles per aggregate as well as the statistical size distribution of the aggregates obtained from the literature.^23^

Figure 3c–e shows a mathematical two-particle model developed by Kendall et al.^11^ that describes the formation mechanism of CB aggregates. In their study, Kendall et al. investigate the contact area between two spherical CB primary particles that belong to two different aggregates. They calculate the necking diameter, given the surface energy γ, the primary particle diameter *D*, Young’s modulus *E*, and Poisson’s ratio ν, as shown in eq 1.
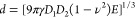
1

On the basis of this two-particle model as well as the number of CB primary particles in an aggregate, the shape and structure of the agglomerates can be calculated (Figure S4). Details of the simulation and modeling techniques are described in the Supporting Information S1.

**Figure 4:**
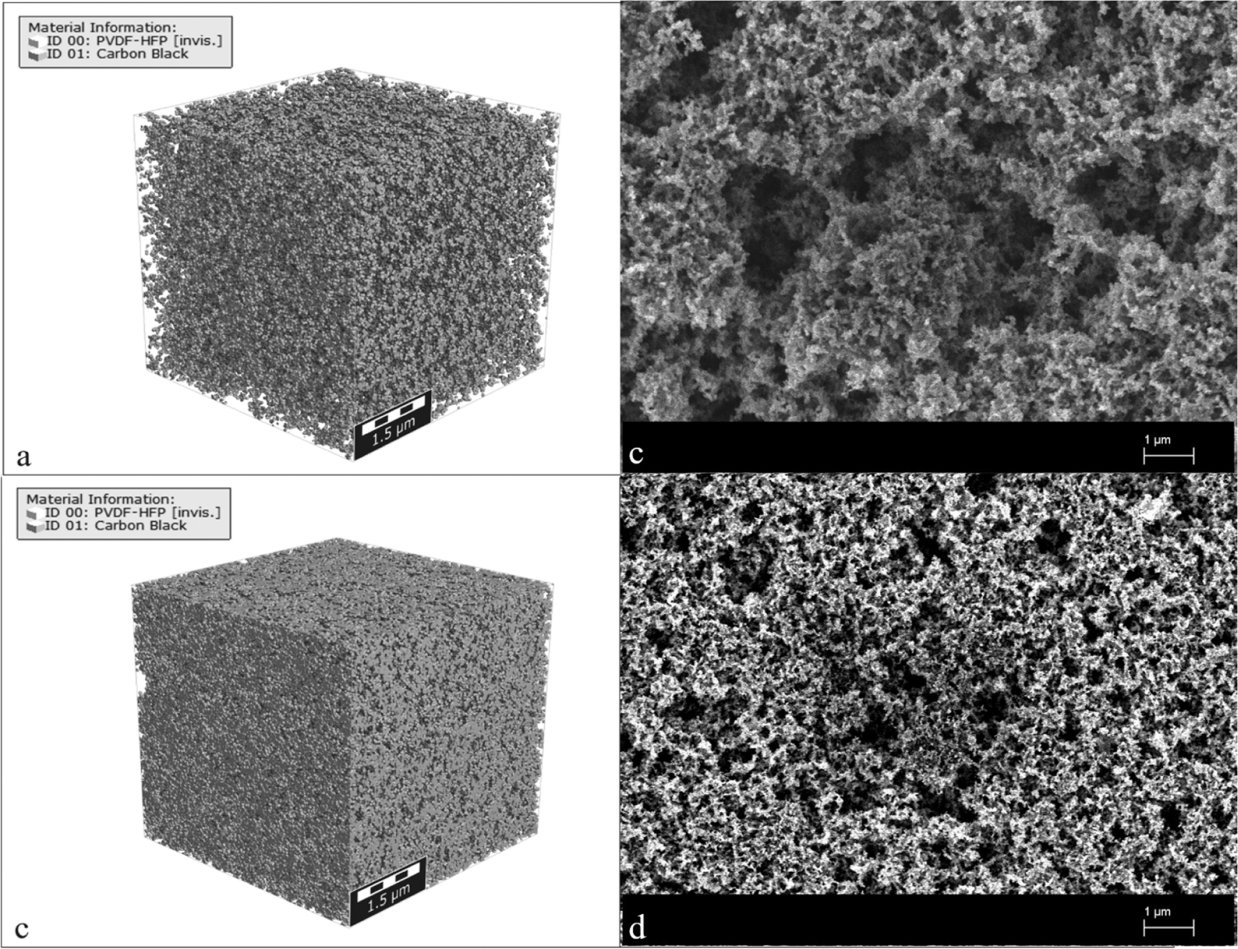
(a) 3D microstructure modeling of a CB-polymer composite (10 vol % CB content). (b) SEM image of 10 vol % CB-polymer sample. (c) 3D microstructure modeling of a CB-polymer composite (30 vol % CB content). (d) SEM image of 10 vol % CB-polymer sample.

After calculating the shape and structure of the aggregates and agglomerate, they are then distributed in the simulated volume, and their position and radius are translated into a voxel-based structural model. The aggregates can be uniformly or anisotropically distributed in the simulated volume to resemble either mechanical mixing (leading to an ordered structure) or a solution-based mixing process (leading to an isotropic distribution of particles). Since a solution-based approach is preferred whenever a conductive additive is introduced during electrode fabrication, we decided to use an isotropic aggregate distribution^16^ to resemble the experimental microstructure of the investigated conductive polymer phase. Figure 4 shows CB distribution within the polymer matrix. At lower CB concentrations, the aggregates and agglomerates tend to distribute homogeneously within the space, while higher CB content leads to the formation of a network structure within the polymer matrix. This correlates well with the scanning electron microscope images of the CB-polymer composite (Figures 4 and S7).

### Calculating the Electrical Conductivity: Numerical FVM Simulation vs DEM Simulation

#### Numerical FVM Simulation Using Ohm’s Law

Next, a three-dimensional conductivity simulation was carried out on the composite matrix which contains both the polymer and the aggregates. A specific material conductivity is assigned to both materials. A potential is applied along the z-axis, and the respective local conductivity is calculated according to eqs 2 and 3.
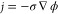
2
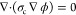
3

Equation 2 is Ohm’s law; eq 3 is the Poisson equation, or, more precisely, the Laplace equation. *j* is the current density in A m^–2^, σ is the effective electrical conductivity in S m^–1^, ϕ is the electrical potential, and σ_c_ is the local electrical conductivity in S m^–1^. The specifics of the simulation are explained in detail in Supporting Information S2.

For solving eqs 2,3 the electrical conductivities for each material must be known. The conductivity value of the polymer is expected to lie between 10^–8^ and 10^–10^ S m^–1^. Due to the polymer’s insulating properties, this value has little to no impact on the calculation.^24−26^ In contrast, graphitic materials have conductivity values ranging from 1 to10^6^ S m^–1^,^18,27−33^ stemming from their morphological structure and the anisotropic conductivity of graphite along or perpendicular to its honeycomb structure. This large range in values for the graphitic conductivity has an immense impact on the conductivity of the resulting composite. To adequately simulate this effect, a carbon black conductivity of 4000 S m^–1^ was assumed.^11^ This value was chosen based on a value published by Kendall et al.,^11^ despite the fact it is one order of magnitude higher (10–500 S m^–1^) than other previously published values for carbon black.^34−36^ The value given by Kendall et al. was obtained from compressed pyrolitic graphite, and for their measurements, they considered only the internal resistance inside a single particle (intra-particle resistance) and omitted the inter-particle resistance between two different particles.

When solving a problem based on the finite volume method, the size of the representative volume element (RVE) as well as the meshing voxel size play an essential role. The RVE size was determined based on the SEM image of the CBP. In the case of low CB content, which causes a more inhomogeneous distribution of CB within the structure, a larger RVE is required. Detailed information about the RVE is provided in Supporting Information S2.

The voxel size influences the smoothness of the geometry and, therefore, the calculation result. Thus, multiple simulations of the same CBP microstructure with different voxel sizes were carried out. These results show that the voxel size should not be larger than 1:10 of the CB primary particle diameter; otherwise, the deviation becomes non-negligible. Detailed information of the voxel size is given in Supporting Information S2.

Using the explicit-jump immersed interface method,^18,27−33^ a fast Fourier transformation (FFT)-based iterative algorithm, the computation time for calculating the conductivity of the composite could be reduced significantly. Periodic boundary conditions were employed for the discretization of eq 3.

#### DEM Simulation Using Kirchhoff’s Law

Based on the same morphological structure and material properties, the electrical conductivity of the bulk material can also be investigated using a mesh-free method. The conductive binder phase can be thought of as an electrical network made up of

(i) inter-particle resistances, which represent the resistances between the particles.
(ii) intra-particle resistances, which represent the resistances inside the particles themselves.

**Figure 5:**
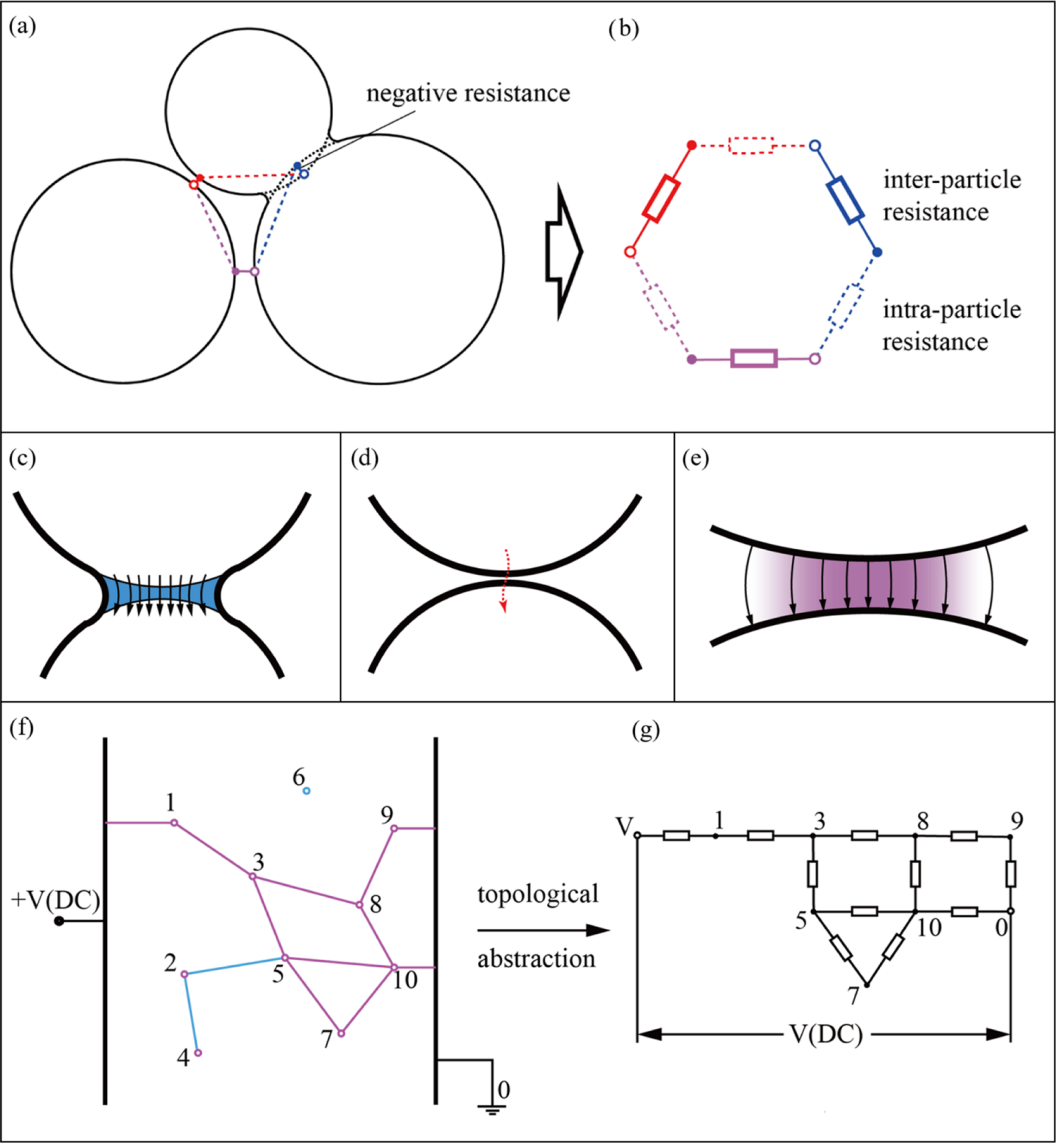
(a) A CB primary particle distribution is abstracted as (b) an electric network topology comprised of inter-particle and intra-particle resistances. (c–e) Inter-particle resistance due to (c) inter-particle bridging, (d) tunneling effect, and (e) an extremely thin layer of polymer that can be regarded as conductive. The black arrows represent the electrical displacement. (f) CB morphological structure and (g) the resulting topological abstraction.

As shown schematically in Figure 5a,b, the geometric arrangement of the CB primary particles can be abstracted as an electric network comprised of inter-particle and intra-particle resistances. The distance between the surfaces of two particles is mainly responsible for the inter-particle resistance, whereas the size of the particles mostly dictates the intra-particle resistance. The calculation of the inter-particle and intra-particle resistances was performed as follows.

The inter-particle resistance is influenced by three effects shown in Figure 5c–e:

(c) Necking between particles, which reduces inter-particle resistance.
(d) The tunneling effect between very close particles, in which particles comprising aggregates show a lower resistivity even without a direct physical connection.
(e) The resistance caused by a polymer layer between two particles, which is so thin that it does not interfere with conductivity.

Geometrically, the inter-particle resistance can be approximated as a cylinder between particles, the diameter and length of which are functions of the inter-particle distance and the particle size. The resistivity of this resistance is dependent on the type of this resistance (necking, tunneling effect, or polymer layer). In this manner, the inter-particle resistances are determined.

For the intra-particle resistance calculation, the Laplace equation (Ohm’s law) is used to represent the distribution of the electrical potential ϕ within the spherical particle (eq 3), whereas eq 2 describes the current density *j* derived by the potential field E. Utilizing these two partial differential equations, the intra-particle resistance as well as the conductivity can be derived. The boundary conditions of eqs 2 and 3 depend on contacting angle(s) between two or multi-particles. Thus, with the geometrical structure information, every intra-particle resistance can be derived. Detailed information is provided in Supporting Information S3.

Ultimately, the large number of inter-particle resistances and intra-particle resistances that act as resistors within the carbon black/polymer composite can be abstracted as a topological network composed of junctions and branches, as depicted in Figure 5f,g. The branches, marked in pink, represent the percolation path through which the current flows, in other words, where the current is not equal to zero. Then, according to Kirchhoff’s law, the effective resistance of the composite can be calculated. A thorough description of the calculation process for the intra-particle resistance is provided in Supporting Information S4.

### Result Discussion and Model Comparison

**Figure 6:**
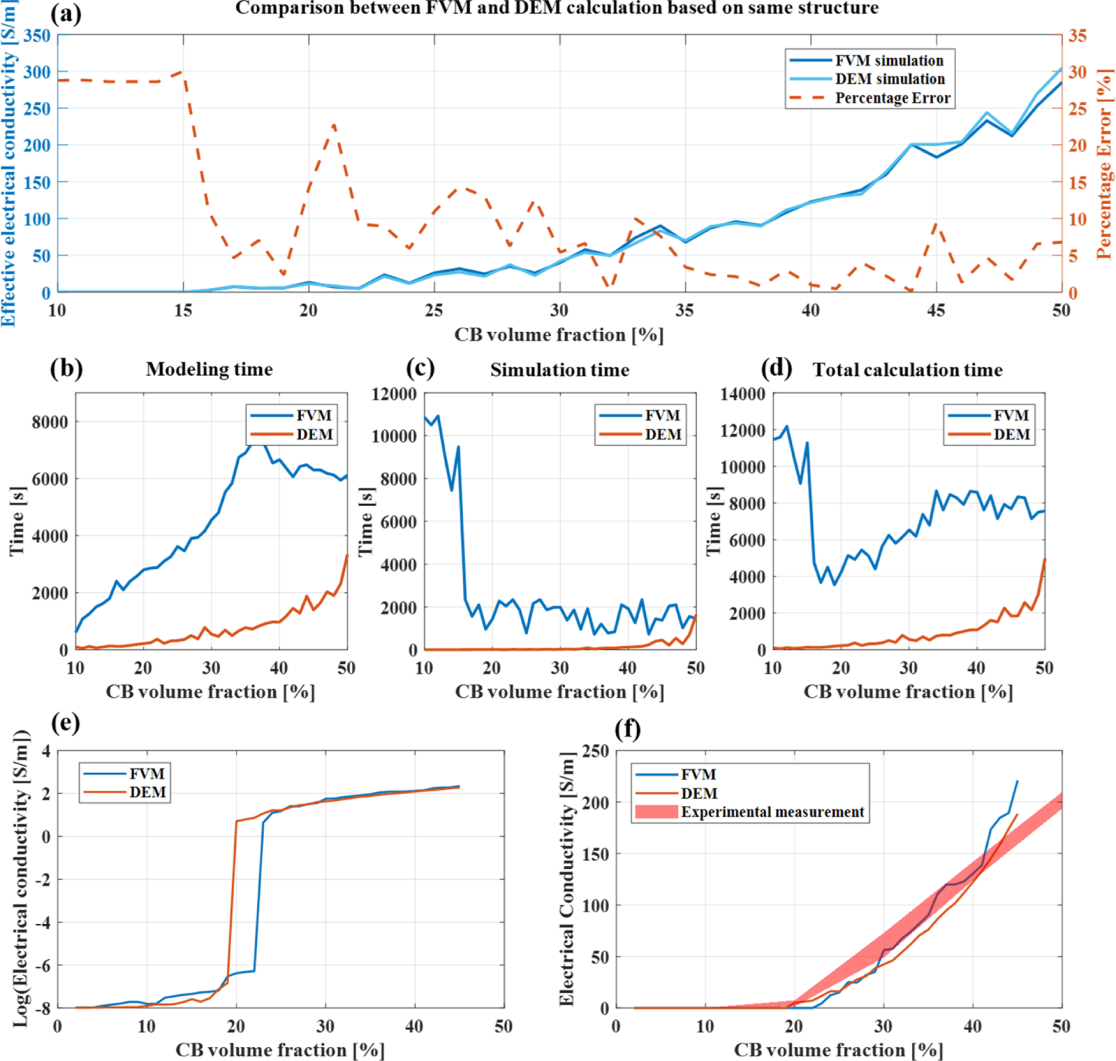
(a) Comparison of the two computational methods DEM and FVM calculating identical microstructures; (b) modeling time; (c) simulation time; (d) total calculation time; (e) electrical conductivity on logarithm axis; and (f) comparison between simulation result and experimental result.

In a first step, identical structures varying in CB content (from 10 to 50%) were simulated with FVM and DEM, with the purpose of validating and checking the accuracy of the DEM model. The calculated results (see Figure 6a) reveal that the simulation of identical structures produces highly consistent results. Figure 6b–d contains the modeling and simulation times. The modeling times of DEM and FVM simulations increase exponentially with an increase in the CB volume fraction. The modeling time for FVM simulations takes much longer than DEM simulations since it is based on an accurate voxel system comprised of a 3-dimensional numerical matrix with a size of 1000 × 1000 × 1000 voxels. An FVM simulation takes approximately 15–35 min on structures with a high CB vol %. In contrast, it takes much longer to get a convergent result at low CB vol %. For DEM simulations, the simulation time also increases with the CB volume fraction. The main advantages of the discrete element method (DEM) over the finite volume method (FVM) are the faster calculation speed for simulating CB volume fractions between 0% and 50% and the fact that DEM simulations require less computation power to achieve the same accuracy.

To determine the volume threshold value of the percolation pathway construction, FVM and DEM simulations of several different microstructures containing the same CB volume fraction were carried out. The FVM and DEM simulation results for the same CB volume fraction were averaged. These results, presented on a logarithmic scale in Figure 6**e**, reveal that when the volume fraction of CB particles reaches around 20%, electrical conductivity is significantly improved. This value is defined as the percolation threshold.^37^

At the very beginning of the percolation transition range—where the composite is only just starting to undergo an insulator-to-conductor transition and electrical conductivity has just increased slightly—a non-ohmic condition is caused mainly by the barrier-tunneling effect in the polymer layers between the distributed carbon black additives.^38^ At this stage, calculations that are performed using FVM or DEM as the modeling method are very similar. Once a larger number of percolation pathways has been formed, the statistical effect of a single percolation pathway on the overall conductivity becomes far less pronounced. Percolation takes place once the content of the conducting additive exceeds the percolation threshold, and the percolation condition transitions from non-ohmic to ohmic.^38^ Near the percolation threshold, conductivity grows exponentially in proportion to the carbon black concentration. Increasing carbon black concentrations will also inevitably reduce the surface distance, a parameter that has a great impact on the non-ohmic state. A slight increase in CB concentration around the percolation threshold substantially enlarges the electrical network, resulting in much improved electrical conductivity. Both the DEM and the FVM simulations can accurately calculate the percolation threshold. Above the percolation threshold, cohesion between the conductive additives in the polymer matrix takes place and a CB network forms. This allows the current to flow more freely (being conducted directly through the linked particles of the conductive additive). During this phase, the electrical conductivity and the CB volume fraction show a linear correlation. A multitude of percolation pathways is formed. Increasing the amount of CB particles further leads to a linear increase in the number of percolation pathways which, in turn, results in a linear increase in the electrical conductivity.

The results from the simulations and the experimental measurements are shown in Figure 6f. The results from the FVM and DEM simulations calculating the electrical conductivity in samples with a CB content between approx 20 −40 vol % correlate very well with the experimental results. This validates both the modeling as well as the computational methods. Larger relative deviations of the electrical conductivity are observed below 20 vol % CB content. This is related to the experimental measurement technique. If the electrical resistance of the sample is too high, the measured voltage reaches the upper limit of the source meter, resulting in a large relative error.

**Figure 7:**
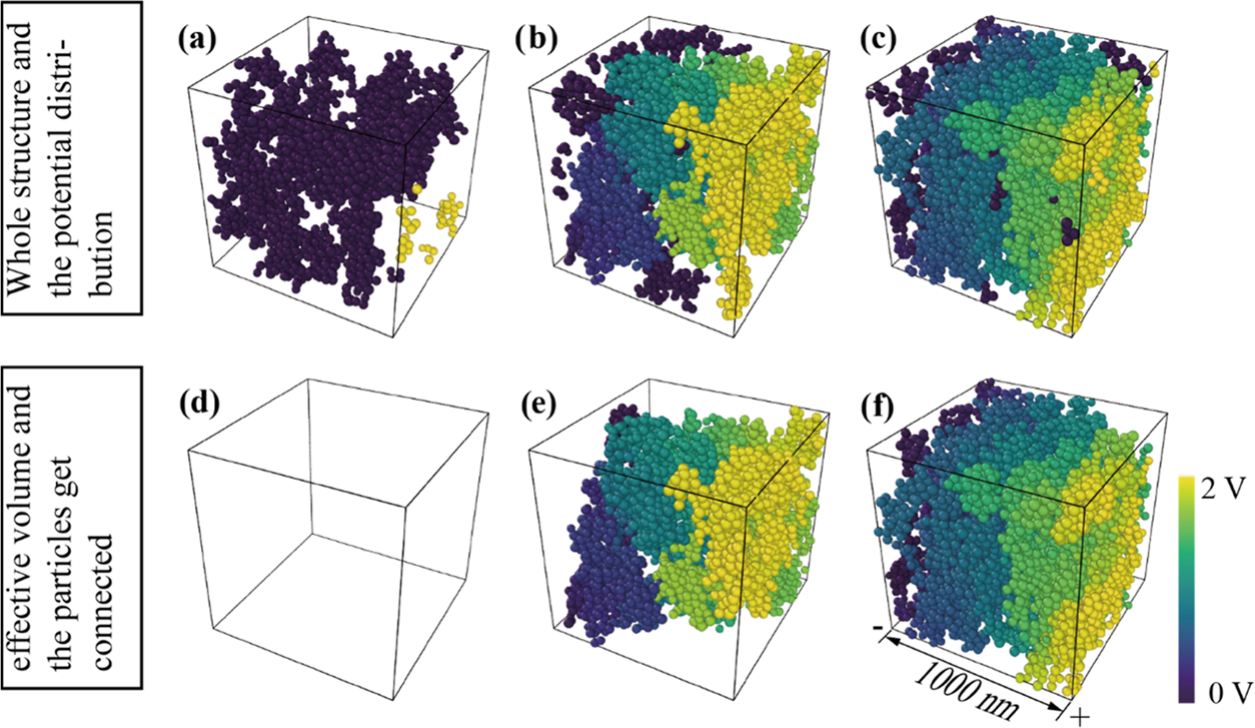
(a–c) DEM simulations of the potential black carbon particle gradient and their connectivity in CB-polymer composite microstructures containing either 10, 20, or 30 vol % carbon black particles; (d–f) the corresponding effective volume between the anode and cathode.

Apart from using DEM simulations, we also visualized the potential CB particle gradient and the connectivity between particles (shown in Figure 7). Figure 7a shows a microstructure matrix with 10 vol % CB particles. Only the particles at the boundary have voltage, whereas the other particles remain unconnected. This indicates a lack of effective connection between the CB particles in the microstructure (Figure 7d). In the microstructure matrix with 20 vol % CB particles (Figure 7b), the electrical network is able to bridge the entire distance from anode to cathode; however, there are also a large number of particles which are not contributing to the electrical network, indicating that they are not connected to it (Figure 7e). Figure 7e depicts the percolating CB particles which are distributed throughout the sample, indicating that a percolation path is established; Figure 7c,f shows a microstructure matrix with 30 vol % CB particles. Nearly all of the particles are effectively connected with each other, revealing that this composite is above the percolation threshold. All in all, DEM simulations are able to visualize the formation of the percolation pathway and reveal that at the very beginning of the percolation transition range, the transition from insulator to a conductive composite is initiated by the formation of one or only a very few effective percolation pathways.

**Figure 8:**
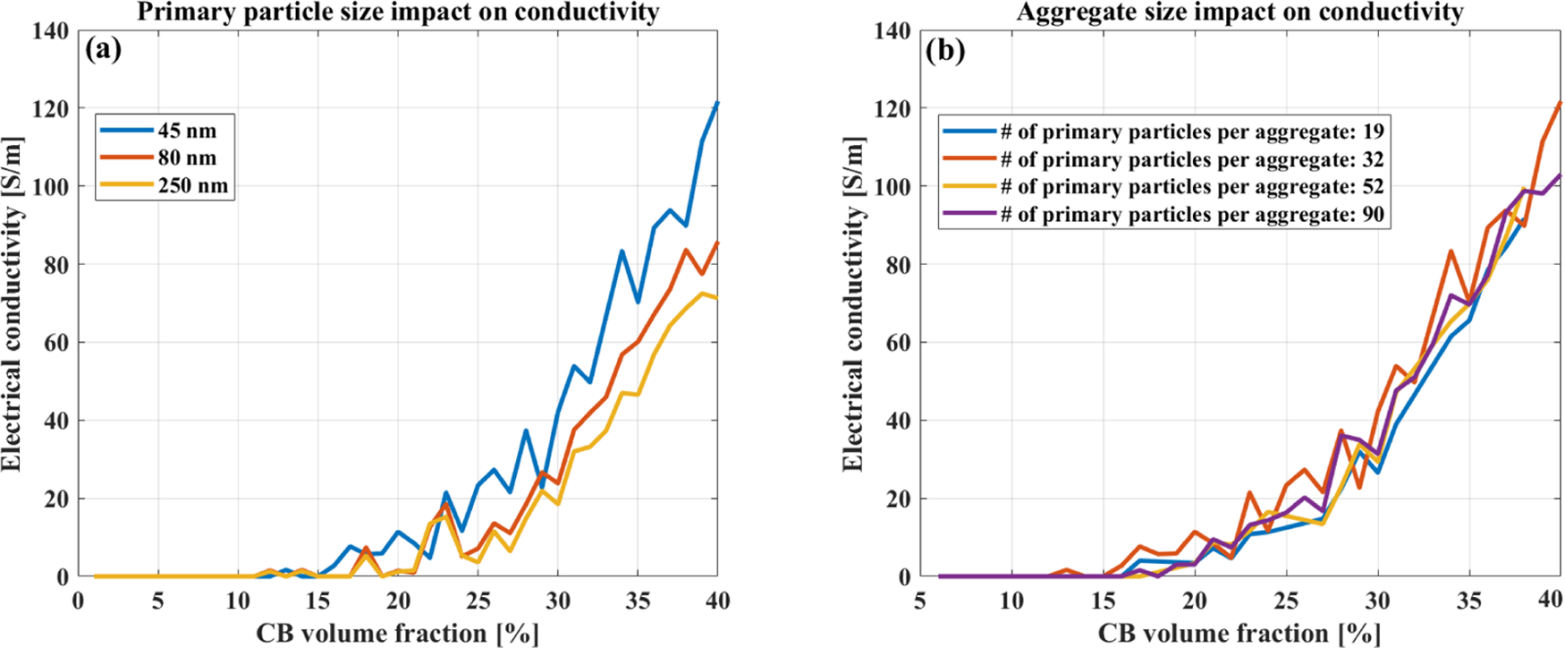
Matrix conductivity as it relates to (a) the average primary particle size, (b) the average size of the CB aggregates.

After successfully validating the accuracy of both the FVM and DEM simulations, we next investigated the impact of the size of CB primary particles and CB aggregates on the electrical conductivity (see Figure 8). Here, we have used exemplarily a Gaussian distribution for CB primary particle size distribution, but any other distribution type can be implemented as well. The aggregate size distribution follows a log-normal distribution;^23^ here, we implement the aggregate size distribution of four different types of carbon black. Because the primary CB particles are much smaller and, therefore, more particles are involved in a certain volume percentage (compared to larger primary particles), which makes it easier to form a CB network structure so that a smaller volume percentage is enough to form a percolation path. As a result, the effective conductivity is higher than in a matrix comprising larger primary particles (compare Figure 8a). In addition, the electrical conductivity of composite microstructures with larger CB primary particles is characterized by a significant fluctuation near the percolation threshold of 20 vol % carbon black content. This indicates that the conductivity is more dependent on the particle distribution than in the case of a microstructure comprising small primary particles. Besides, in industry, due to variations in the way the CB is produced, the aggregate size distribution varies for different types of carbon black. To account for these effects, we varied the average number of primary particles per aggregate, distributed them in the polymer matrix, and then ran the simulation. These results, shown in Figure 8b, reveal no significant influence of the aggregate size on the effective electrical conductivity. The reason is: on the one hand, larger aggregates tend to form spherical shape compared to small aggregates, which causes an ineffective usage of CB particles to form a percolation path; on the other hand, larger aggregates mean more primary CB particles are strongly fused together, which benefits the overall electrical conductivity. These two effects cancel each other out and lead to this result.

In summary, our simulation results validated with experimental results can be utilized as a comprehensive template on how to design a CB-polymer composite for a wide range of different applications, such as for example, the conductive binder phase of lithium-ion batteries. For the application in batteries, a volume fraction of CB higher than 30 vol % is needed to achieve conductivity in the range of 180–220 S m^–1^, a fact that is significant for overall battery design and, specifically, the production of the conductive binder phase. Moreover, smaller CB primary particles are necessary to provide good connectivity between active material particles.

## Conclusions and Outlook

The study quantitively investigates the electrical conductivity of CB-polymer composites through detailed modeling of the aggregates and subsequent simulation of the electrical conductivity. The simulations provide a specific and reliable guide for CB-polymer composite material design. First, we mathematically depicted the mechanism of how CB particle aggregates and agglomerates are formed. In a second step, we proceeded to model this process based on both finite volume method (FVM) and discrete element method (DEM) simulations of the electrical conductivity. We were able to achieve a significant improvement in the computation time for the DEM simulations. We also achieved a very good correlation between the two simulation methods as well as experimental results. The obtained results are in good agreement with other experimental studies investigating the conductive binder phase,^3,8^ which determined the appearance of percolation paths at around 20–30% volume fraction carbon black.

Different types of composites—other than the investigated carbon black/polymer system—can also be investigated using this technique. In the future, the addition of conductive additives with complex shapes (e.g., carbon nanotubes, graphene) will become increasingly important. When adding these, their anisotropic shape as well as their interaction with the surrounding matrix and their distribution in the matrix will need to be considered. This study provides the necessary tools for these evaluations.

The methodology described in this work can also be adapted to study the thermal conductivity of a wide range of materials and composites. Especially in cases where the addition of additives like copper, silver, or gold particles is common, such as in the production of thermally conductive paste or food containers with porous thermal isolation, this methodology could be hugely helpful. For further insights into these possibilities, it will be necessary to simulate a wide range of different types of aggregates. Besides, in some practical applications, a significant volume change of the conductive polymer may impact the percolation path and cause deagglomeration of aggregates,^39^ which is worthwhile to be investigated and included into our study in the future.

## Supporting Information Available

S1: van der Pauw method for electrical conductivity measurement, carbon black spatial distribution modeling; S2: FVM-based simulation; S3: DEM-based simulation; S4: coupling of the inter-particle and the intra-particle resistances (PDF)

## Abbreviations

CB: carbon black
CBP: conductive binder phase
DEM: discrete element method
FVM: finite volume method
RVE: representative volume element
