# Supplementary material for: Microstructural Modeling and Simulation of a Carbon Black-Based Conductive Polymer—A Template for the Virtual Design of a Composite Material
Source: ACS Omega. 2022 Aug 11;7(33):28820–30. doi: 10.1021/acsomega.2c01755 (PMC9404170; doi:10.1021/acsomega.2c01755)
Supplement: Supplementary file 1 — ao2c01755_si_001.pdf [file ao2c01755_si_001.pdf]

Microstructural modeling and simulation of a carbon black-based conductive polymer – a template for the virtual design of a composite material

Yuanzhen Wang[1,2], Chensheng Xu[3], Timotheus Jahnke[1,2], Wolfgang Verestek[3], Siegfried Schmauder[3], Joachim P. Spatz[1,2]

[1] MAX PLANCK INSTITUTE FOR MEDICAL RESEARCH, DEPT. OF CELLULAR BIOPHYSICS, Jahnstraße 29, 69120 Heidelberg, Germany

[2] HEIDELBERG UNIVERSITY, INSTITUTE FOR MOLECULAR SYSTEMS ENGINEERING (IMSE), Im Neuenheimer Feld, 69120 Heidelberg, Germany

[3] UNIVERSITY OF STUTTGART, INSTITUTE FOR MATERIALS TESTING, MATERIALS SCIENCE AND STRENGTH OF MATERIALS (IMWF), Pfaffenwaldring 32, 70569 Stuttgart, Germany

Supporting Information S1

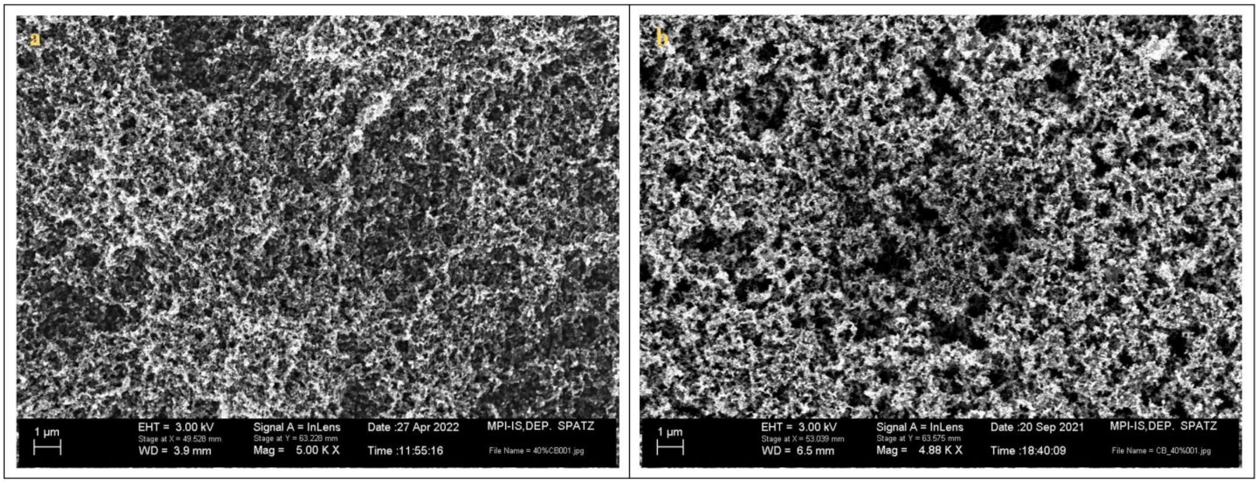

Figure S1. SEM images of CB-polymer differing in polymer: a) PVDF-HFP; b) CMC.

Van der Pauw method for electrical conductivity measurement

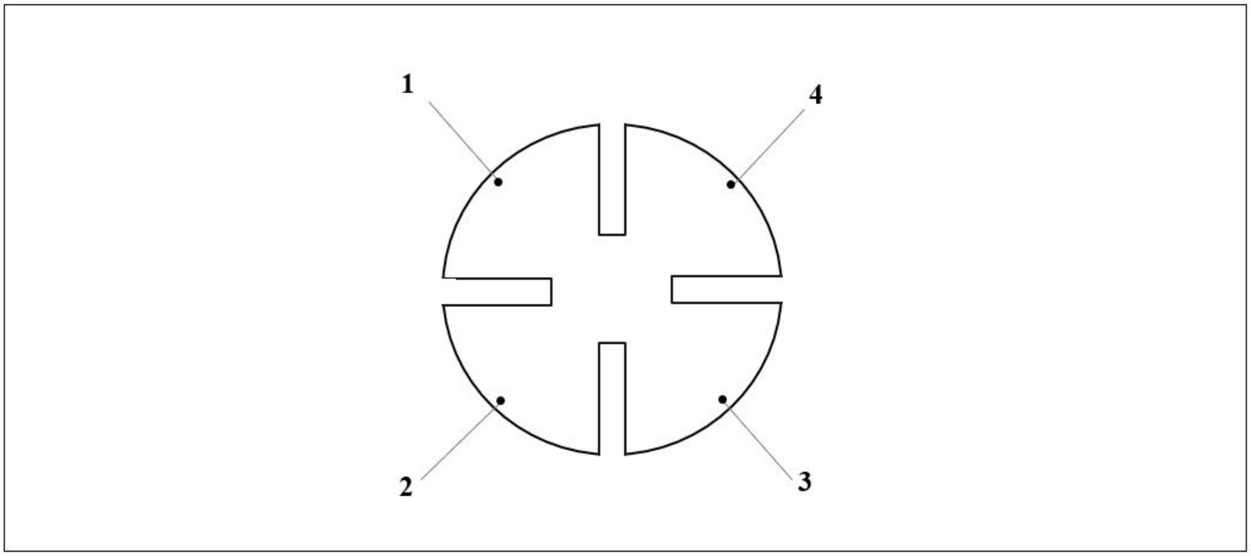

Figure S2. Clover leaf-like shaped sample used for van der Pauw measurements.

Carbon black spatial distribution modeling

Aggregate formation

The aggregate formation workflow is shown in **Figure S3**. The aggregation process is built on the MATLAB platform since a large number of numerical matrix calculations are needed during this phase. The collision between CB primary particles as well as the surface growth during production were simulated based on this modeling algorithm. In the early phase of production, free particle movement and particle collision happen abundantly. During this phase, the network backbone of the aggregate starts to form. This was modeled by randomly selecting a particle from the existing structure, assigning a new primary particle with a collision angle, and letting the latter connect to the structure. Surface growth slows down later during production which was modeled by increasing the radius of primary particles and letting them overlap with each other.

It is worthwhile mentioning that, in order to reduce the computational effort, all the primary particles were modeled based on a polar coordination system. When the aggregation process was finished, all positions of CB primary particles were transformed into a Cartesian coordination system. Large numbers of aggregates can be modeled based on this algorithm, so that an aggregate database can be constructed during this step. This database can then be used to model the entire structure of the CB-polymer composite in a voxel-based system using Geodict®.

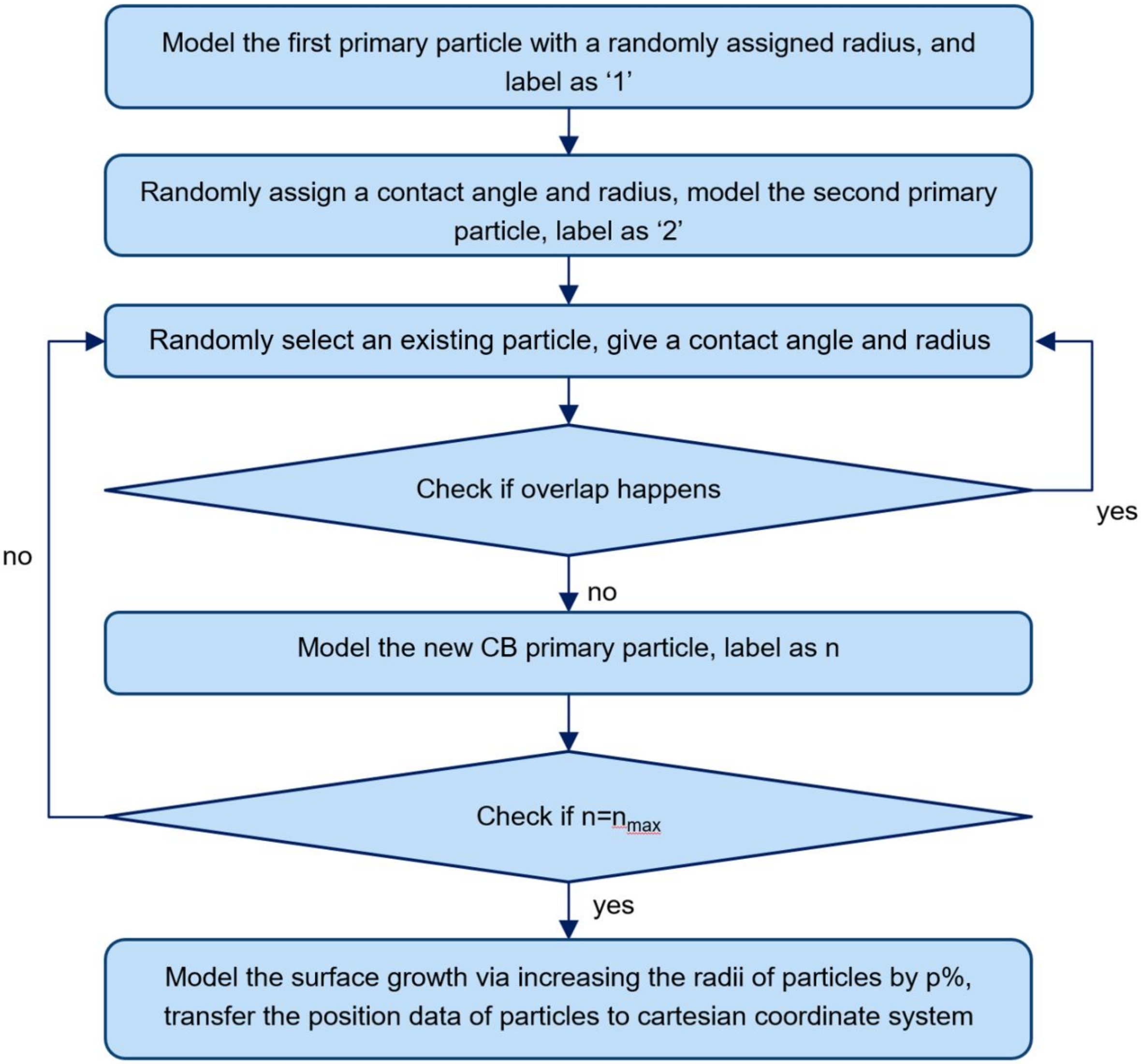

Figure S3. Workflow of single CB aggregate modeling.

According to the type of CB and polymer that is used, aggregation and agglomeration can vary. The described algorithm also provides a high flexibility for responding to relevant parameters, such as CB primary particle size distribution, the extent of surface growth, the number of primary particles per aggregate, and more.

When modeled aggregates were compared to real aggregate structures (see **Figure S4**) it was revealed that various structures, both linear and clustered, were modeled successfully in this step.

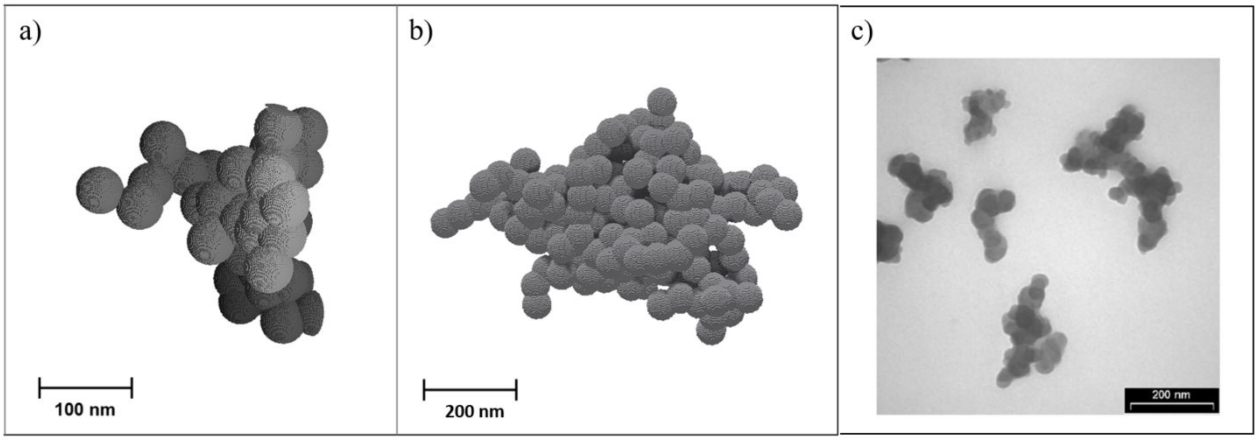

Figure S4. Comparison of modeled and real CB aggregates. a) The modeled structure containing 30 primary particles; b) The modeled structure containing 100 primary particles; c) TEM image of CB aggregates.

Aggregate distribution

For the distribution of these aggregates in a voxel-based system, first, a random selection of aggregates from the aggregate database was chosen. Aggregates that contain different numbers of primary particles were selected based on the log-normal distribution. Around the surface of the aggregates there is a thin layer where particles commonly overlap (shown in **Figure S5**). The thickness of this thin layer was determined using **Equation 1**. Next, selected aggregates were randomly assigned a position in the polymer matrix. If there was a collision in the form of overlap between these aggregates, a series of small random translation vectors as well as small random rotation angles were assigned to this aggregate until the aggregate fit into the available space (the workflow for this process is shown in **Figure S6**). CB aggregate distribution was modeled using Geodict®.

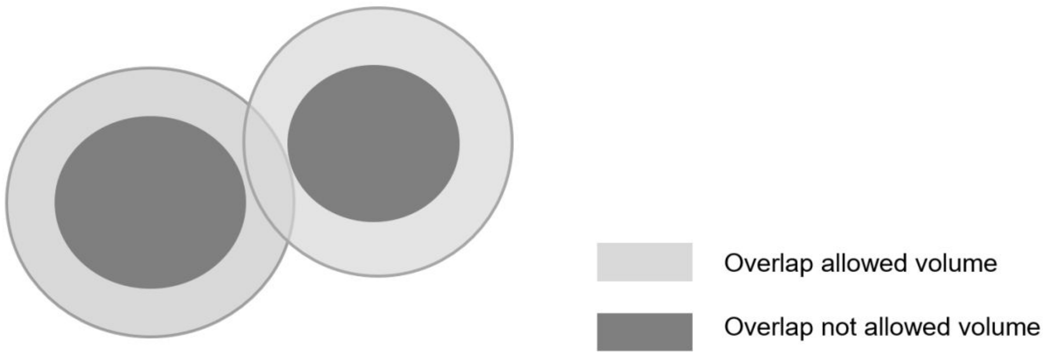

Figure S5. Two CB primary particles from two different aggregates are allowed to overlap only to a certain extent. The maximum allowed overlapping volume was calculated using **Equation 1.**

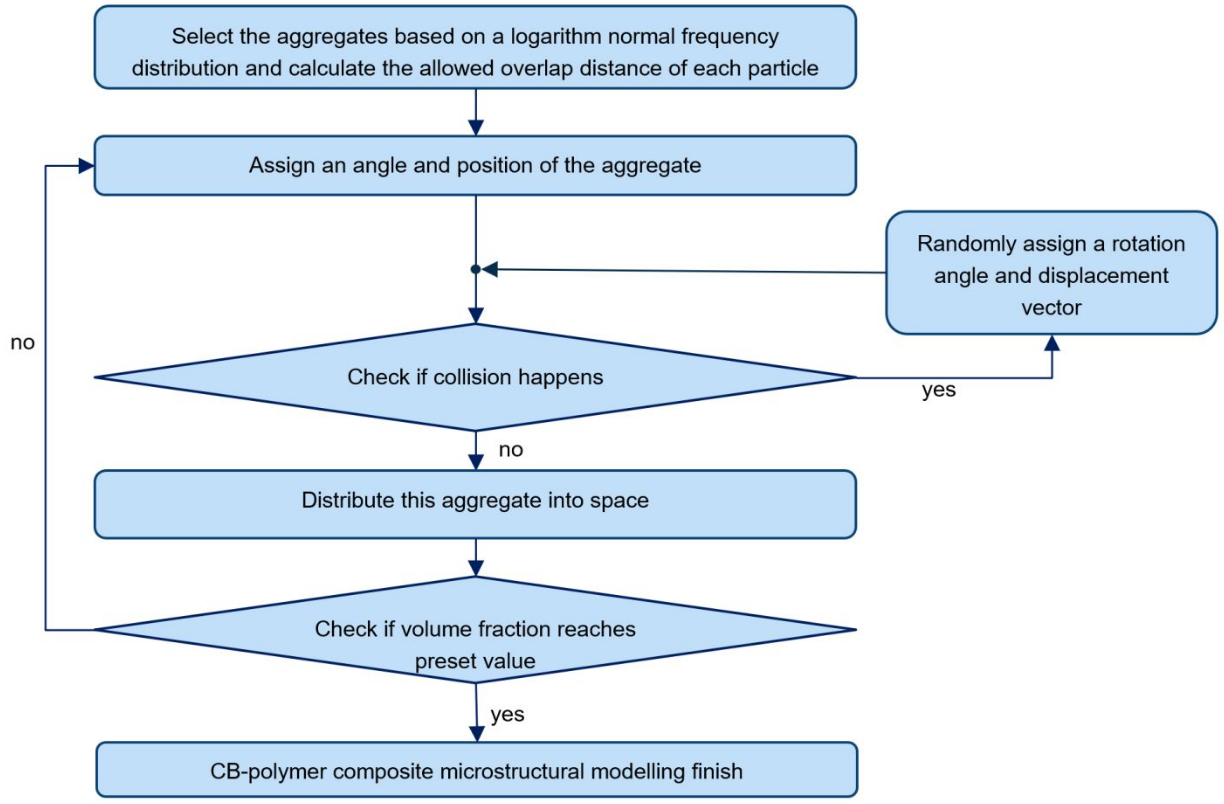

Figure S6. Workflow for modeling the spatial distribution of CB aggregates.

The modeled CB-polymer composite structure is shown in **Figure 4** in the paper, which shows great similarity to actual scanning electron microscopy images (**Figure S7**). When the CB content in the polymer matrix is low, the aggregates tend to distribute isotopically in the available space. Increasing the CB content leads to more connections between aggregates as they form agglomerates and network structures.

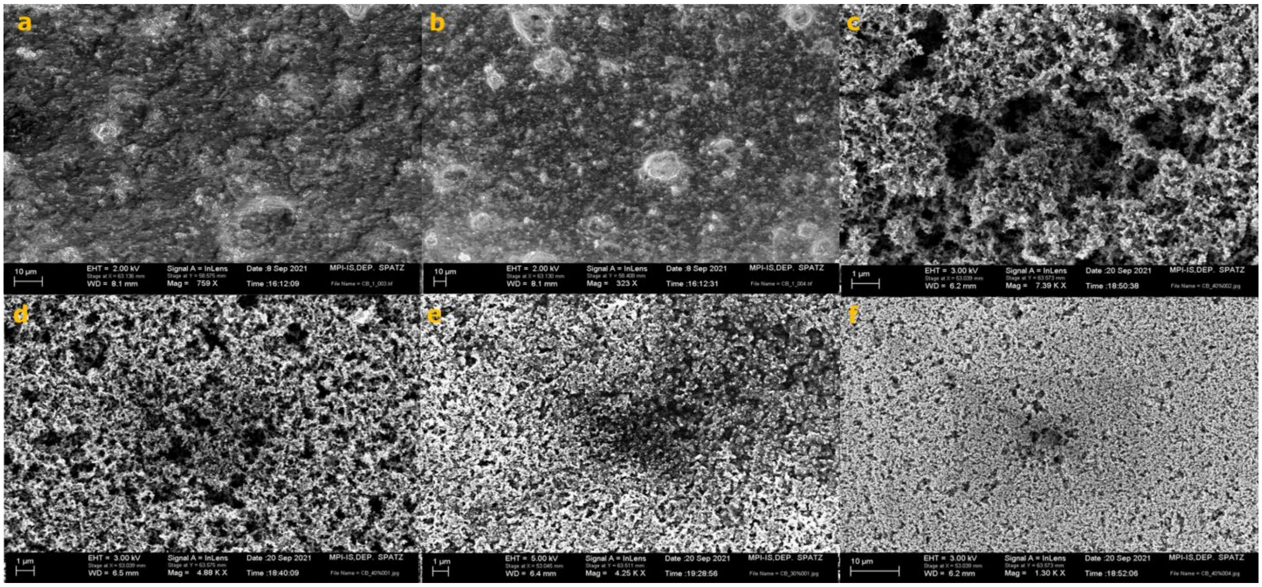

Figure S7. SEM images of CB-polymer composites differing in carbon black content: a) 5 vol.%, b) 10 vol.%, c) 20 vol.%, d) 30 vol.%, e) 40 vol.%, and f) 50 vol.% CB.

Supporting Information S2

FVM-based simulation

Solver, convergence criteria and boundary conditions

FVM calculations were based on the Explicit-Jump Immersed Interface Method which solves the elliptic differential equation at the interface where the solution is not smooth. An iterative method is used: starting with an initial guess at the solution, an iteration – a repetitive computational cycle of operations – is carried out to approximate the current values more closely until the stopping criterion is reached. A tolerance value is preset to detect if the iterative process becomes stationary. If the change in the electrical conductivity value between iterative steps is smaller than the tolerance, the process is stopped. **Equation S1** defines the tolerance value. In our case, the tolerance value was set to 0.0001.

$$Tolerance = \frac{(new\ value\ - previous\ value)}{previous\ value} \qquad \textbf{Equation S1}$$

In addition, a periodic boundary condition, which assumes that the structure is repeated periodically in the space, is applied.

Representative volume element and voxel size

The representative volume element size is obtained by studying the microscopic morphology of CB-polymer composites. As **Figure S7** shows, the structure of the composite becomes more homogeneous as the CB volume fraction increases. A larger RVE is needed to get a reliable result for low CB volume fractions. Therefore, as a compromise between computational accuracy and computational effort, a 5 µm × 5 µm × 5 µm RVE was set.

Because the voxel size has an impact on the interface smoothness, it also influences the calculation results. A convergence study was carried out by calculating the effective electrical conductivity on the same microstructure with differently sized voxels. The results show that when the voxel size is smaller than 1/10 of the CB primary particle, the calculation result deviations are less than 5%. Considering that the diameter of all the primary particles is larger than 20 nm and their average diameter is 45 nm, the voxel size was set at 2 nm.

Supporting Information S3

DEM-based simulation

The Discrete Element Method (DEM) using Python as the programing language presents an alternative method for modeling the morphology of the agglomerates and for calculating the conductivity of the composites, especially in view of the many particle interactions that take place (such as overlapping, bridging and electric conductance). After performing the convergence study, the RVE size for DEM simulations was set at 2 μm × 2 μm × 2 μm.

Modeling section

DEM modeling mainly focuses on central positions and the radius of each spherical particle. The approach is very similar to the one mentioned in the **Supporting Information S1**.

Figure S8a shows the first step in the simulation process: Newly generated candidate particles (referred to as child particles) are assigned a contact or controlled overlapping with an existing parent particle in the agglomerate. The relative position of the child is based on its parent utilizing a Monte Carlo method, a method that makes use of a random number generator. However, if there is an overlapping volume that exceeds the set threshold maximum volume value with any other particle in the space, the program will generate a new candidate child particle. Voxels at higher levels, which most likely correspond to connected components of the original, are labeled as roots. **Figure S8a)** illustrates this process. **Figure S8b)** shows how the calculation effort was reduced. When testing whether there is a collision between the child of the agglomerate originating from root1 and the agglomerate originating from root2, only the surface distance of particles in the suspected area (yellow outlined particles in the gray ring) are checked.

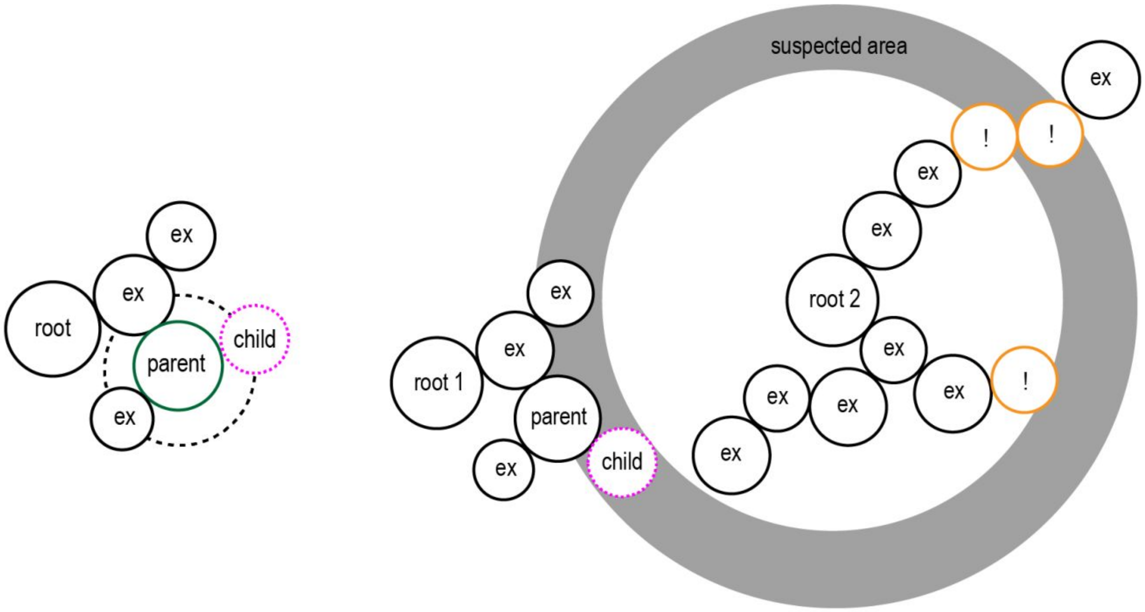

a) **b)**

Figure S8. a) New (child) particles must have contact with the surface of their parent; **b)** Only the particles that have contact with the suspected area (marked as a gray ring) are scrutinized in the collision check.

If two particles overlap due to the aggregates surface growth during the CB production phase or agglomeration during dispersion, an interparticle bonding neck is created between two CB primary particles. The shape of two overlapping particles during the forming of an aggregate/ agglomerate is shown in **Figure S9**. The blue part is referred to as the interparticle bonding neck and the yellow part is the overlapping volume. Both volumes are considered equal to each other in order to maintain the volume consistency.

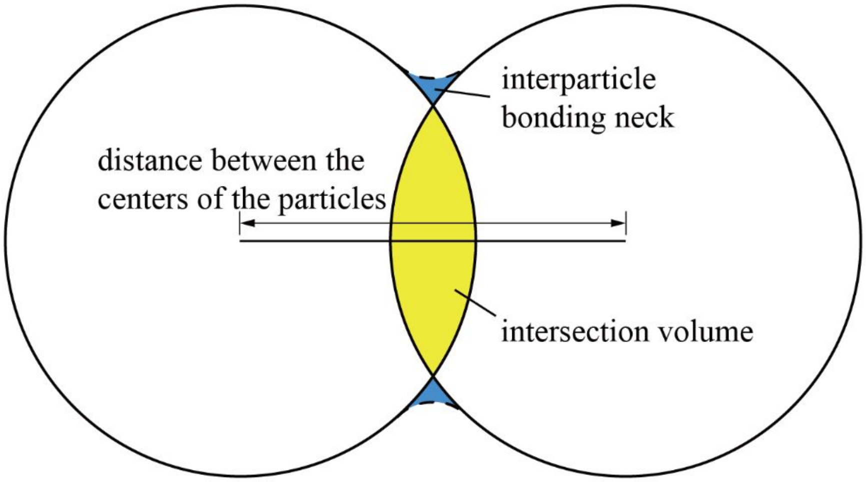

Figure S9. Interparticle bridging. The volume of the yellow intersection volume (overlap) is considered equal to the volume of the blue interparticle bonding neck.

Simulation section

DEM abstracts the geometrical morphology of CB-polymer composite to an electrical circuit, on the purpose of calculating the electrical conductivity of the material. The CB-polymer composite can be thought of as an electrical network made up of:

(i) inter-particle resistances, which represent the resistances between the particles;

(ii) intra-particle resistances, which represent the resistances inside the particles themselves.

1. Calculation of the inter-particle resistance

Figure S10 shows the relationship between the surface and the inter-particle resistance. The inter-particle resistance curve can be divided into four phases[1]. Every phase has its physical meaning and a different algorithm is used to calculate the relationship during each phase. A detailed description of each phase is given below.

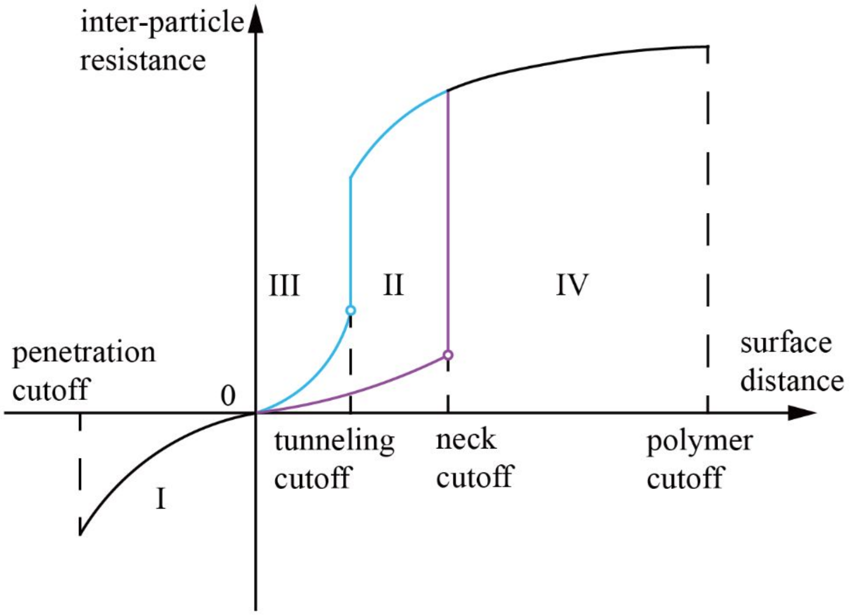

Figure S10. The relationship between inter-particle resistance and the surface distance between two particles.

I. Inter-particle overlap/penetration

As the particles come into contact, penetrate, and form a collective, the combined conductivity improves. To account for this increase in conductivity, the intra-particle resistance should be calibrated by adding the negative value of the "negative" inter-particle resistance[2].

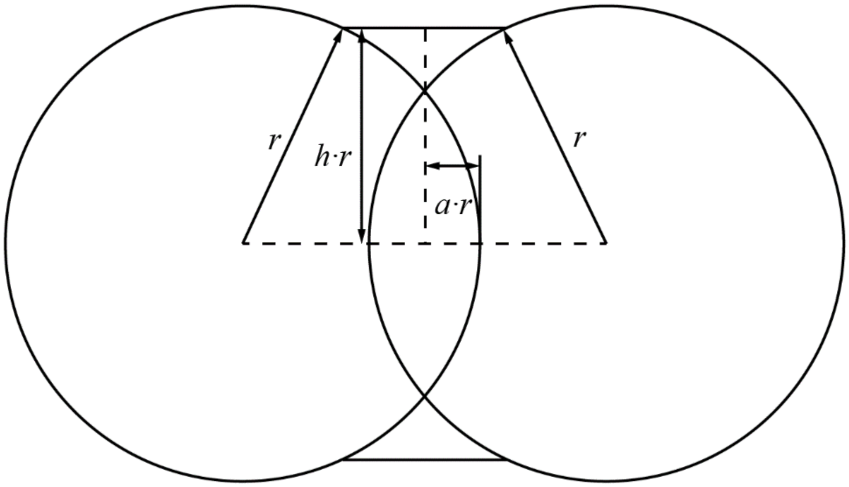

Figure S11. Calculating the circular area of the interparticle bonding neck.

Figure S11 shows the parameters required for calculating the inter-particle resistance during the penetration phase: r_{sphere} refers to the particles´ identical radium, $a \cdot r$ is the overlap distance, and $h \cdot r$ can be calculated with the geometrical derivate:

$$-\frac{1}{3}h\left(\left(\frac{1}{h}-\sqrt{\frac{1}{h^2}-1}\right)^2\left(\frac{3}{h}+\sqrt{\frac{1}{h^2}-1}-1\right)-\frac{3}{h}+3\sqrt{\frac{1}{h^2}-1}\right)=a$$ **Equation S2**

To achieve a higher computational efficiency, the implicit expression can be approximately fitted to the explicit expression resulting in

$$0.66\ln(100 \cdot a+1)=h$$ **Equation S3**

The inter-particle resistance during the penetration phase calculated as

$$R_{inter} \approx \frac{0.02(1-a)}{((0.2+0.66\ln(100 \cdot a+1))\sigma r_{sphere})} - 0.103 \cdot {}^{1}/\sigma r_{sphere}$$ **Equation S4**

II. Neck growth by surface diffusion

Analogously, the volume of the neck can be calculated. It can be assumed that the neck, which is formed by surface diffusion, is formed by a diffusion volume that is a volume fraction β of the sphere volume. Therefore, the neck size, as it relates to the surface distance d, can be estimated as:

$$r_{neck} = \sqrt{\frac{\beta \cdot \frac{4}{3} r_{sphere}^3}{d}} \qquad \textbf{Equation S5}$$

When calculating r_{neck} we assumed that the diffusion will not influence the original surface distance of the sphere by more than 10%. β was solved as 27.1%.

The inter-particle resistance during the neck-growth phase was calculated as

$$R_{inter} \approx \frac{d^2}{0.271\sigma \cdot \frac{4}{3}\pi r_{sphere}^3} \qquad \textbf{Equation S6}$$

III. Tunneling effect

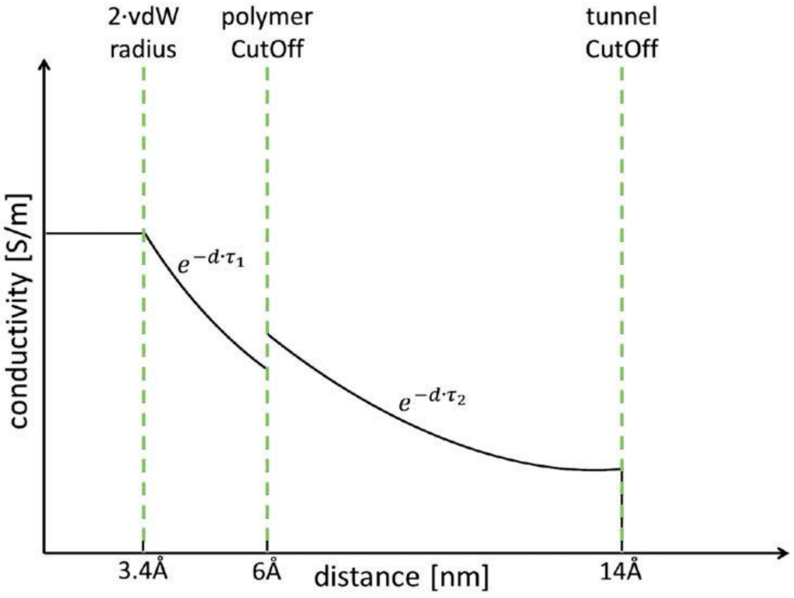

Figure S12. Schematic representation of the dependency of the conductivity on the spheres' surface distance. The graph shows a maximum conductivity value for distances below or equal to the van-der-Waals radius as well as two exponential decays in the curve and a negligible value for non-tunneling distances**Error! Reference source not found.**.

Figure S12 shows the conductivity, which is the inverse of the resistance, between the particles as it relates to the surface distance of the spheres which was calculated as

$$\sigma(d) = \begin{cases} e^{-\tau_1 \cdot 3.4}, & d \in (0, 0.34]nm \\ e^{-\tau_1 \cdot d}, & d \in (0.34, 0.6)nm \\ e^{-\tau_2 \cdot d}, & d \in [0.6, 1.4)nm \end{cases}$$ **Equation S7**

The barrier 1 conductivity τ_1 and barrier 2 conductivity $\tau_2$were 22.9 nm^{-1} and 17.7 nm^{-1}, respectively.**Error! Reference source not found.**

IV. Ohmic polymer conductivity

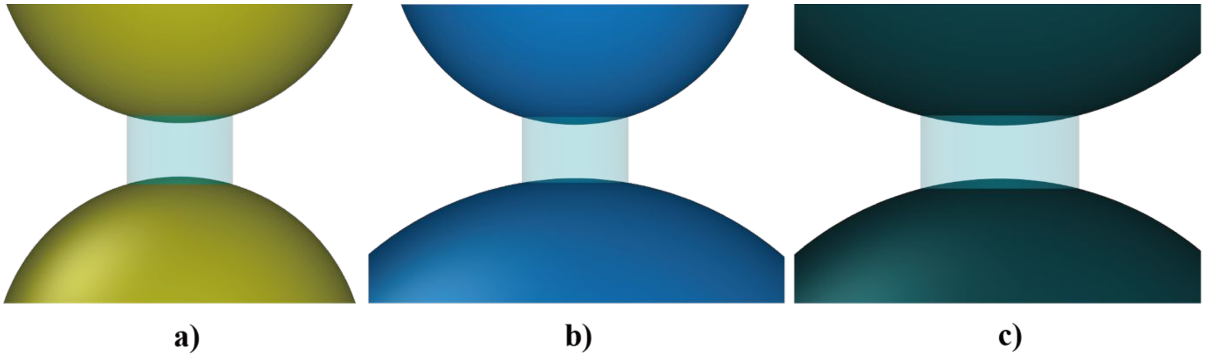

Figure S13. The size of the smaller particle in the combined pair determines the size of the contact area for ohmic polymer conduction. a) conductive polymer between two small particles; b) conductive polymer between one small and one big particle; c) conductive polymer between two big particles.

During this phase, each particle acts as a conductive node in a large number of serial and/or parallel resistances constituting the electrical network. The resistance between two particles (i.e. nodes) consists of the inherent resistance of the spherical particle multiplied by its radius and the resistance of the surrounding polymer multiplied by the distance between the two particles[3] (see **Figure S13**).

The inter-particle contact resistance can be calculated using the size of the smaller sphere through integrating the polymer resistance between the spherical surfaces:

$$\frac{1}{R_{inter}} = -\ln\left(\left|\frac{d}{2 \cdot R_{min}}\right|\right) \cdot \sigma_{poly} \cdot 2\pi \cdot R_{min} \qquad \textbf{Equation S8}$$

2. Calculation of the intra-particle resistance

First, we looked at the case when the contact is directly on the two poles of the sphere**Error! Reference source not found.** (as shown in **Figure S14**).

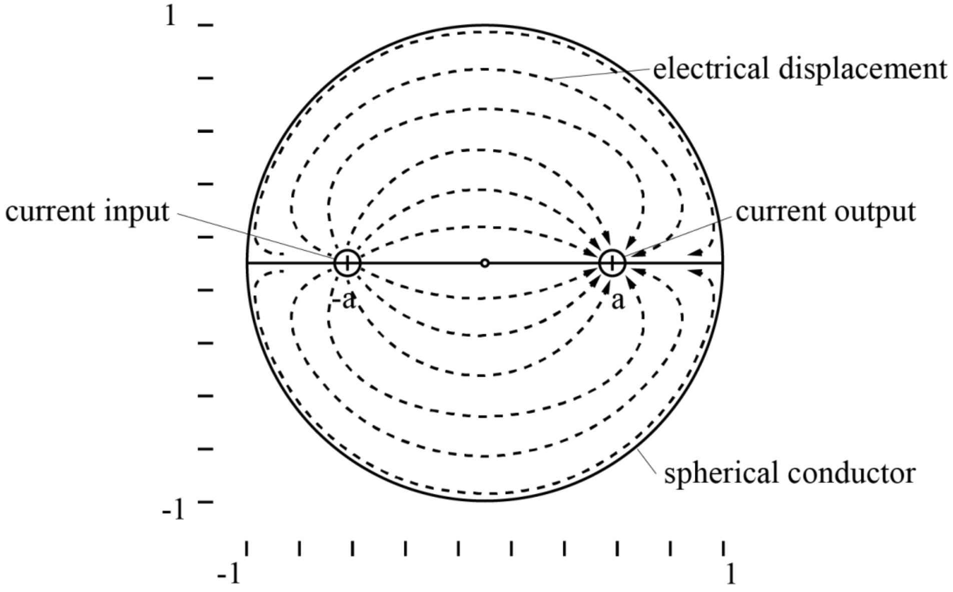

Figure S14. The electrical displacement when the input and output currents are located at $(-a,0,0)$, $(a,0,0)$.

When the input and output currents are located at $(-a,0,0)$, $(a,0,0)$ and the radius of the sphere equals 1, the conductivity σ is also normalized. Using the axle symmetric spherical coordinate, the distribution of the potential ϕ can be expressed with the axis symmetrical coordinate (r, θ):

$$\nabla^2\phi = -\delta(r-a)[\delta(\cos\theta+1) - \delta(\cos\theta - 1)]/2\pi r^2 \qquad \textbf{Equation S9}$$

After dealing with the function in two different solution ranges, $r \in (a,1)$ and $r \in (0,a)$, they can be connected on the boundary.

The integral was then approximated with discrete summary statistics. The trial function given below should fulfill the boundary conditions, because c_n, d_n, $P_n(\cos\theta)$ represent trial factors:

$$\phi(r,\theta) = \begin{cases} \sum_{n=0}^{\infty} c_n\left((n+1)r^n + \frac{n}{r^{n+1}}\right)P_n(\cos\theta), & r \in (a,1) \\ \sum_{n=0}^{\infty} d_n r^n P_n(\cos\theta), & r \in (0,a) \end{cases}$$ **Equation S10**

On the shared boundary, we obtained

$$c_n\left((n+1)a^n + \frac{n}{a^{n+1}}\right) = d_n a^n$$ **Equation S11**

and

$$\left(n(n+1)c_n\left(a^{n+1} - a^{-n}\right) - n d_n a^{n+1}\right)P_n(\cos\theta) = \frac{\delta(\cos\theta+1) - \delta(\cos\theta-1)}{2\pi}$$ **Equation S12**

These can be solved using the trial integer m:

$$c_{2m-1} = -\frac{a^{2m-1}}{2\pi(2m-1)}$$ **Equation S13**

$$d_{2m-1} = -\frac{1}{2\pi}\left(\left(1 + \frac{1}{2m+1}\right)a^{2m+1} + a^{-2m}\right)$$ **Equation S14**

We used the generating function to define the Legendre polynomials:

$$\sum_{n=0}^{\infty} P_n(x)t^n = \frac{1}{\sqrt{1-2xt+t^2}}$$ **Equation S15**

We solved the infinite series up to the sum

$$\phi(r,\theta) = \frac{1}{4\pi}\Bigg(\frac{1}{\sqrt{1+2ar\cos\theta+(ar)^2}} - \frac{1}{\sqrt{1-2ar\cos\theta+(ar)^2}} + \left(\frac{1}{\sqrt{a^2+2ar\cos\theta+r^2}} - \frac{1}{\sqrt{a^2-2ar\cos\theta+r^2}}\right) - \ln\left(1-ar\cos\theta + \sqrt{1+2ar\cos\theta+(ar)^2}\right) + \ln\left(1-ar\cos\theta+\sqrt{1-2ar\cos\theta+(ar)^2}\right)\Bigg)$$

After calculating the general expression of the potential ϕ, we set $a\rightarrow1$ to obtain:

$$\phi(r,\theta) = \frac{1}{4\pi}\Bigg(\frac{2}{\sqrt{1+2r\cos\theta+r^2}} - \frac{2}{\sqrt{1-2r\cos\theta+r^2}} - \ln\left(1-r\cos\theta+\sqrt{1+2ar\cos\theta+(r)^2}\right) + \ln\left(1-r\cos\theta+\sqrt{1-2r\cos\theta+(r)^2}\right)\Bigg)$$

We calculated the input and output potential at the contact area when $r=1$, $\cos\theta = \pm(1-\delta\theta^2/2)$:

$$\phi = \pm\frac{1}{2\pi\delta\theta}$$ **Equation S16**

Using the general Ohm's law, the resistance $R_{equivalent}$ was calculated with the conductivity σ:

$$R_{equivalent} = \frac{r_{sphere}}{\pi\sigma r_{contact}\cdot r_{sphere}} = \frac{1}{\pi\sigma r_{contact}}$$ **Equation S17**

where $\delta\theta$ means the ratio between the contact area radius and the sphere radius.

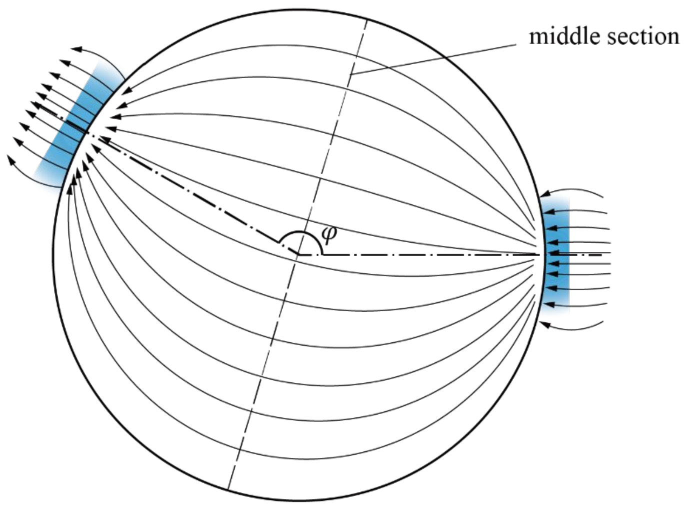

Figure S15. When the two different particle contacts are not symmetric to the axis of the sphere, it is necessary to assume the angle of the contact to calculate the respective intra-particle resistance.

Next, we looked at the more common case when two contacts are not on the axis of the sphere (**Figure S15**). Here, we approximated the resistance to the effective sphere in the complete sphere. When the contacts lie at an angle φ to the axis of the sphere, the intra-particle resistance was expressed as:

$$R_{equivalent}(\varphi) = \frac{1}{\pi\sigma r_{contact} \cdot \sin\frac{\varphi}{2}}$$ **Equation S18**

To calculate the intra-particle resistance, we assumed the values are evenly distributed in the range of $\varphi \in (\alpha \cdot \pi, \pi)$, we set $r_{contact} = 0.2 \cdot r_{sphere}$ to obtain an assumedly flat surface, and α is the shape factor related to the shape distribution and how advanced the penetration between the 3-body system is:

$$\overline{R_{equivalent}} \approx 0.103 \cdot {}^{1}/\sigma r_{sphere}$$ **Equation S19**

Supporting Information S4

Coupling of the inter-particle and the intra-particle resistances

Geometrically, the inter-particle resistance can be approximated as a cylinder between particles, the diameter and length of which are functions of the inter-particle distance and the particle size. The cylindrical resistance is calculated according to **Equation S19**. The resistivity value ρ varies based on the effect of the different influences shown in **Figure 5c - e**.

$$R = \frac{\rho \cdot l}{S} = \frac{4\rho \cdot l}{\pi r d^2} \qquad \textbf{Equation S20}$$

In addition, a cut-off distance ($\frac{R_{min}}{10}$) value is set. This value describes the threshold value above which the distance between two particles is too large to form an effective electrical connection.

For the intra-particle resistance calculation, the Laplace equation (Ohm's law) is used to represent the distribution of the electrical potential ϕ within the spherical particle (**Equation 3**), whereas **Equation 2** describes the current density j derived by the potential field E. Utilizing these two partial differential equations, the intra-particle resistance as well as the conductivity can be derived (**Supporting Information S3**). As for the boundary condition, it depends on the contact points (schematically shown in **Figure S16a**). To solve the Laplace equation inside the particle and get

the effective resistance between the two contact points, boundaries for the inlet and outlet potential – ϕ_{inlet} and ϕ_{outlet} , respectively – were placed at the contact points. For a more complex situation with multiple contact points (more than two) on one spherical particle, an electric network topology was established (shown in **Figure S16b**). However, the intra-particle resistances (R_{intra_1}, R_{intra_2} and R_{intra_3} in **Figure S16b**) separately influence the path they belong to because they are not connected to each other. For example, when calculating the resistance between node 1 and node 2, we simply compute the sum of R_{inter_1}, R_{intra_1} and R_{inter_2}. Neither R_{intra_2} nor R_{intra_3} are a part of this calculation, because they are not connected to the electric flow.

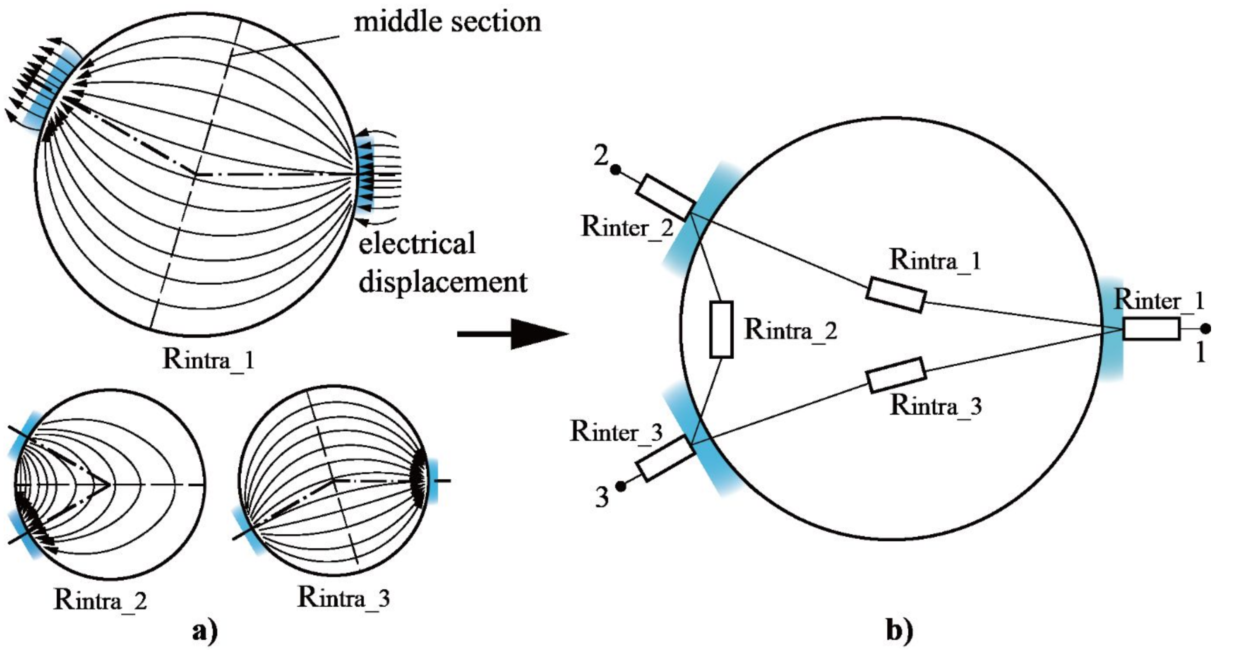

Figure S16. a) CB particle with two contact points: the position of the points determines where to set boundary conditions for calculating the effective resistance; **b)** For calculations on particles with more than two contact points a network is constructed.

Then, the intra- and inter-particle resistances are able to form an electrical circuit. As depicted in **Figure 5f - g**, the branches, marked in purple, represent the percolation path through which the current flows, in other words, where the current is not equal to zero. Then, according to Kirchhoff's law, the effective resistance of the composite can be calculated.

References

(1) Penazzi, G.; Carlsson, J. M.; Diedrich, C.; Olf, G.; Pecchia, A.; Frauenheim, T. Atomistic Modeling of Charge Transport across a Carbon Nanotube–Polyethylene Junction. *J. Phys. Chem.* **2013**, *117* (16), 8020–8027.

(2) Smythe, W. B. Static and Dynamic Electricity. 1988.

(3) Bartels, J.; Jürgens, J.-P.; Kuhn, E.; Ploshikhin, V. Effects of Curvature and Alignment of Carbon Nanotubes on the Electrical Conductivity of Carbon Nanotube-Reinforced Polymers Investigated by Mesoscopic Simulations. *J. Compos. Mater.* **2019**, *53* (8), 1033–1047.
